# Single-cell imaging of *N*^4^-acetylcytidine-modified RNA using fluorine metabolic labeling mediated proximity ligation assay

**DOI:** 10.1093/nar/gkaf464

**Published:** 2025-06-04

**Authors:** Qi Wang, Yuhao Du, Shen Yan, Ziang Lu, Yongling Tang, Feng Xiao, Fuling Zhou, Xiang Zhou

**Affiliations:** Department of Hematology of Zhongnan Hospital, Taikang Center for Life and Medical Sciences, Wuhan University, Wuhan 430071, China; College of Chemistry and Molecular Sciences, Wuhan University, Wuhan, Hubei 430072, China; College of Chemistry and Molecular Sciences, Wuhan University, Wuhan, Hubei 430072, China; College of Chemistry and Molecular Sciences, Wuhan University, Wuhan, Hubei 430072, China; College of Chemistry and Molecular Sciences, Wuhan University, Wuhan, Hubei 430072, China; College of Chemistry and Molecular Sciences, Wuhan University, Wuhan, Hubei 430072, China; Department of Hematology of Zhongnan Hospital, Taikang Center for Life and Medical Sciences, Wuhan University, Wuhan 430071, China; Department of Hematology of Zhongnan Hospital, Taikang Center for Life and Medical Sciences, Wuhan University, Wuhan 430071, China; College of Chemistry and Molecular Sciences, Wuhan University, Wuhan, Hubei 430072, China

## Abstract

*N* ^4^-Acetylcytidine (ac^4^C) is an emerging epitranscriptomic mark involved in regulating RNA stability, translation, and gene expression. Despite its emerging role in gene regulation and disease, current methods for *in situ* detection of ac^4^C-modified RNA lack sensitivity and specificity. To overcome these challenges, we developed fluorine metabolic labeling mediated proximity ligation assay (FMPLA), a method combining fluorine-based metabolic labeling with a proximity ligation assay for precise detection of newly synthesized fluoro-metabolically labeled ac^4^C sites. This approach enables high-sensitivity visualization of multiple RNA species, and provides insights into the abundance and spatial dynamics of ac^4^C-modified RNAs during the cell cycle and under chemotherapeutic stress. Additionally, FMPLA reveals distinct RNA modification patterns in drug-resistant cancer cells, highlighting its potential in studying functions and mechanisms of RNA ac^4^C modification.

## Graphical Abstract

**Graphical Abstract:**
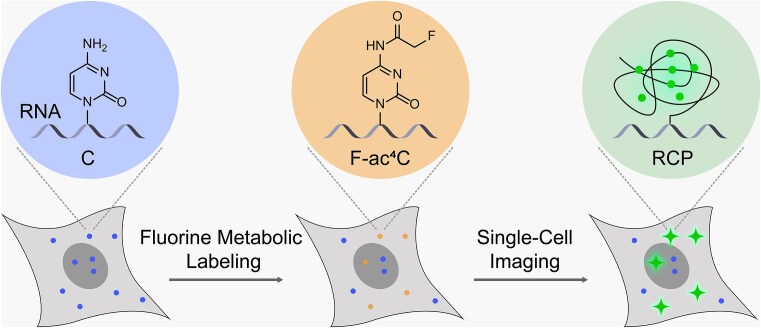

## Introduction

*N* ^4^-Acetylcytidine (ac^4^C) is a novel and relatively rare epigenetic modification in RNA that influences various cellular and biological processes across eukaryotic species, prokaryotic species, and viruses [1]. First identified in yeast transfer RNA (tRNA) in 1966 and later in mammalian tRNA [2–5], ac^4^C was subsequently found in ribosomal RNA (rRNA) [6,7], messenger RNA (mRNA) [8, 9], and non-coding RNAs (ncRNAs) [10, 11], highlighting its widespread presence across various RNA types. Functionally, ac^4^C modification plays an essential role in gene expression regulation by enhancing RNA stability and regulating mRNA translation efficiency [9, 12 ,13]. Dysregulation of ac^4^C has been linked to infections [14–17], autoimmune diseases [18–20], and cancers [21–29], making it a potential factor in disease progression. However, despite growing recognition of its importance, our understanding of how ac^4^C RNA is regulated in physiological and pathological contexts remains limited. Therefore, developing effective methods to detect and study ac^4^C RNA is crucial for elucidating its role in gene regulation and addressing ac^4^C-related diseases.

Several methods have been developed to study ac^4^C modifications. Early techniques, such as post-labeling fingerprinting and rapid gel sequencing, enabled quick and visual detection of ac^4^C modifications but lacked resolution and throughput [5]. High-performance liquid chromatography allowed precise quantitative analysis of ac^4^C but could not pinpoint specific modification sites within RNA sequences [8, 30]. Advanced techniques, such as acRIP-seq [9] (immunoprecipitation and sequencing), utilized next-generation sequencing to map ac^4^C sites across the transcriptome. Methods such as ac^4^C-seq [31] (reduction with sodium cyanoborohydride and sequencing) and RedaC:T-seq [32] achieve single-nucleotide resolution. However, these transcriptome-wide or cell lysate-based methods cannot provide information about ac^4^C in its native cellular context. Techniques that capture epigenetic modifications at the single-cell and single-molecule levels are essential for understanding cell heterogeneity and disease mechanisms [33–37]. Additionally, current methods lack the ability to conveniently target specific RNAs and to image ac^4^C sites in disease-related genes, underscoring the need for a cost-effective and straightforward detection approach.

**Figure 1.**
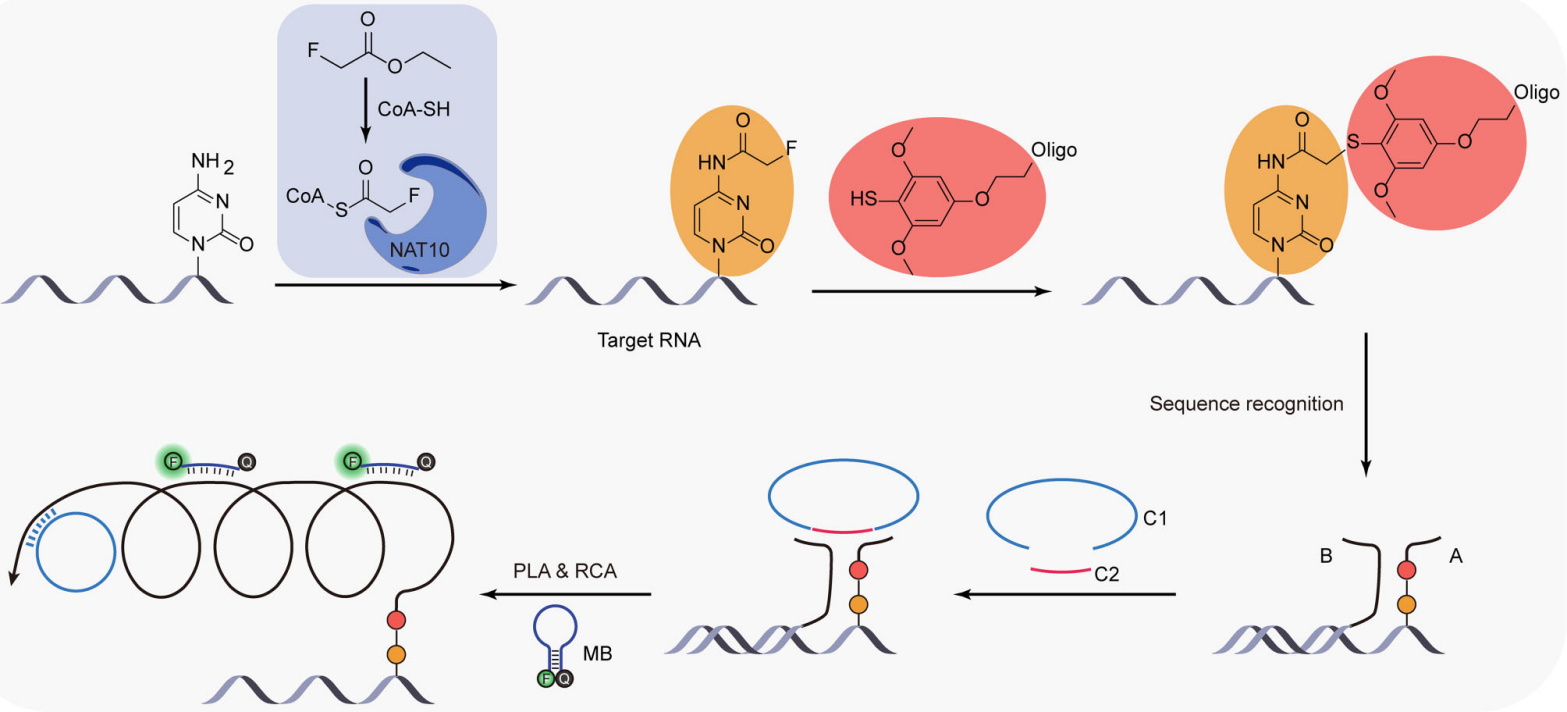
Schematic representation of FMPLA. After treatment with ethyl fluoroacetate, the presumed ac^4^C sites on RNA within cells are metabolically labeled as F-ac^4^C, followed by conjugation with Probe A through a fluorine–thiol displacement reaction (FTDR). Subsequently, RISH is performed with Probe B, which hybridizes to a specific region of the target RNA. Probes A and B, which are spatially adjacent, provide a platform for the ligation of Probe C1 and Probe C2. Upon the formation of the circular template through the proximity ligation, RCA extends Probe A, producing a long amplicon containing repeated sequences. Molecular beacon (MB) then recognizes the complementary sequence on the amplicon, unfolding its hairpin structure and generating a fluorescent signal.

Previously, our group developed an innovative method called FAM-seq (fluorine-assisted metabolic sequencing), an antibody-free method for mapping ac^4^C RNA [38]. FAM-seq uses metabolic labeling to detect newly acetylated RNA sites in cells and allows for subsequent labeling reactions and enrichment sequencing. Building on this, we have developed a robust *in situ* imaging method for ac^4^C RNA, termed fluorine metabolic labeling mediated proximity ligation assay (FMPLA). This method integrates the F-ac^4^C (fluoro-metabolically labeled ac^4^C sites) labeling probe with RNA *in situ* hybridization (RISH) DNA probes. The former selectively conjugates with F-ac^4^C residues, while the latter targets RNA sequences adjacent to ac^4^C sites via sequence-specific hybridization. We utilized a proximity ligation assay (PLA) [39–48], which produces a robust and proximity-dependent signal, thereby minimizing false-positive signals that may arise from detecting individual targets or distant dual targets (Fig. 1). This combination of F-ac^4^C reactive probes and RISH DNA probes ensures high selectivity for ac^4^C RNA. The dual recognition mechanism triggers *in situ* ligation and rolling circle amplification (RCA), producing a strong signal from fluorophore-labeled DNA probes, which results in high detection sensitivity.

After confirming the effectiveness of FMPLA across various cell models and ac^4^C-modified RNA types, changes in ac^4^C RNA abundance were examined at different cell cycle stages. Interphase cells exhibited the highest levels of ac^4^C-modified RNA, with a sharp reduction during the mitotic stages, suggesting a strong link between ac^4^C modifications and cell cycle. In cisplatin-treated models, significant accumulation of ac^4^C-modified ncRNA in nuclear foci was observed, indicating a potential role in cellular stress responses and DNA repair. In K562/ADR cells, ac^4^C RNA levels were downregulated following both short- and long-term chemotherapy treatments, highlighting its possible involvement in drug resistance mechanisms. These findings emphasize ac^4^C’s critical role in cellular processes and its broader implications in disease and therapeutic responses. The FMPLA technique offers significant potential for advancing RNA biology research and its applications in disease models, including cancer, by providing a powerful tool for studying ac^4^C’s functional roles in various biological contexts.

## Materials and methods

### Materials

Supplementary Table S1 provides the oligonucleotide sequences. Supplementary Table S2 lists the types of ac^4^C-modified RNA imaged in this study along with their accession numbers.

### Cell culture and treatments

The human cervical cancer cell line (HeLa), human embryonic kidney 293T cell line (HEK293T), and non-tumorigenic breast epithelial cell line (MCF-10A) were obtained from the China Center for Type Culture Collection. The chronic myeloid leukemia (CML) cell line (K562) and its adriamycin-resistant variant (K562/ADR) were purchased from Cellverse Co., Ltd. HeLa and HEK293T cells were maintained in Dulbecco’s modified Eagle medium (DMEM, Gibco) supplemented with 100 U/ml penicillin, 100 μg/ml streptomycin sulfate (Gibco), and 10% fetal bovine serum (FBS, Gibco) at 37°C in a humidified 5% CO_2_ incubator. MCF-10A cells were cultured in specific medium for MCF-10A cells (Procell Co., Ltd.) under the same conditions. K562 and K562/ADR cells were cultured in Iscove’s modified Dulbecco’s medium (IMDM, Procell Co., Ltd.) supplemented with 10% FBS, 100 U/ml penicillin, and 100 μg/ml streptomycin at 37°C in 5% CO_2_.

For metabolic labeling, cells were seeded in 20-mm glass-bottom dishes and cultured to approximately 70%–90% confluence. The medium was then replaced with DMEM complete medium containing 2 mM ethyl fluoroacetate, and the cells were incubated for 8–12 h at 37°C.

For NAT10 inhibitor treatment, HeLa cells were seeded to reach 70%–90% confluence in 20-mm glass-bottom dishes. The cells were pretreated with 50 μM Remodelin (MedChemExpress, HY-16706) for 16 h at 37°C, followed by incubation with 2 mM ethyl fluoroacetate as described above.

For small interfering RNA (siRNA) transfection, HeLa cells were plated and allowed to adhere for 24 h prior to the transfection of siRNAs targeting CA9 or NAT10 (JTSBIO Co., Ltd.). Transfection was performed using Lipofectamine RNAiMax (Thermo Fisher) according to the manufacturer’s instructions. After transfection, the cells were treated with ethyl fluoroacetate as described above.

Thymidine blocking was performed as previously described [49]. Briefly, HeLa cells were seeded in 20-mm glass-bottom dishes and grown to 70%–90% confluence, and then incubated with complete medium supplemented with 2 mM thymidine for 18 h. The medium was then aspirated, and the cells were washed twice with phosphate buffered saline (PBS) and once with prewarmed medium. To release the cells from the first block, fresh medium was added, and the cells were incubated for 9 h. A second thymidine block was applied by treating the cells with complete medium containing 2 mM thymidine for another 18 h. After the second block, the cells were washed and released as described above. DMEM complete medium supplemented with 2 mM ethyl fluoroacetate was added to the cells 2 or 8 h before the end of the release period.

For cisplatin treatment, HeLa cells were seeded to reach 70%–90% confluence in 20-mm glass-bottom dishes and incubated with 0.5 μM cisplatin (Yeasen) in DMEM complete medium for 16 h at 37°C.

For doxorubicin (DOX) treatment, 5 × 10^5^ K562 and K562/ADR cells were seeded into 20-mm glass-bottom dishes and cultured in IMDM complete medium supplemented with 1 μg/ml DOX (Aladdin, A183027) for 24 h, followed by ethyl fluoroacetate treatment as described above.

### RNA extraction and purification

Total RNA from all samples was isolated using TRIzol (Invitrogen™) according to the manufacturer’s instructions. The concentration of the purified RNA was then measured using a NanoDrop 2000 spectrophotometer (Thermo Fisher).

### RT-qPCR

For the assay, 50 ng of total RNA and 200 nM primers were used with TransScript Green One-Step RT-qPCR SuperMix (TransGen Biotech) following the manufacturer’s recommendations on a Bio-Rad CFX96 Touch™ Real-Time PCR Detection System. Each sample was analyzed in three independent assays. The data were collected using Bio-Rad CFX manager software.

### Western blotting

HeLa cells were washed twice with cold PBS and lysed in 100 μl of RIPA buffer [50 mM Tris–HCl, pH 7.4, 150 mM NaCl, 1.5% NP-40, 0.1% sodium dodecyl sulfate (SDS), 0.5% sodium deoxycholate, 2 mM MgCl_2_] containing 1× PIC (Halt protease and phosphatase inhibitor cocktail, Pierce) at 4°C for 30 min. Lysates were cleared by centrifugation in 12 000 × *g* at 4°C for 30 min. Protein concentration was determined using a Pierce BCA protein assay kit (Thermo Fisher Scientific) according to the manufacturer’s instructions. Equal quantities of protein were mixed with SDS loading buffer, boiled at 95°C for 10 min, and loaded onto SDS–PAGE (polyacrylamide gel electrophoresis) gels. The separated proteins were transferred onto a PVDF membrane (Millipore) in an ice bath at 70 V for 2 h. The PVDF membrane was blocked in TBST (Tris-buffered saline, 0.1% Tween 20) containing 5% (w/v) bovine serum albumin (BSA) (Beijing Dingguo Changsheng Biotechnology Co., Ltd.) at 37°C for 1 h under agitation. Then, the PVDF membrane was incubated with primary antibodies Rabbit anti-GAPDH (Proteintech) or Rabbit anti-NAT10 (Proteintech) overnight at 4°C. The membrane was washed three times with TBST at room temperature, and then incubated with HRP-conjugated Goat Anti-Rabbit IgG (H + L) (Proteintech) in TBST containing 5% (w/v) BSA at 37°C for 1 h. The membranes were washed three times again with TBST at room temperature and imaged on a ChemiDocTM XRS+ Imaging System (Bio-Rad) after incubation with Rhea ECL (US Everbright, Inc.).

### Preparation of ADB–Probe A

4-(2-azidoethoxy)-2,6-dimethoxybenzenethiol (ADB) was synthesized following the method described by Yan *et al.* [38]. The click reaction was conducted at 55°C for 10 min, using 100 μM DBCO-Probe A and 2 mM ADB. Subsequently, the ADB–Probe A conjugate was purified using the Oligo Clean & Concentrator Kit (Zymo Research, D4061) as per the manufacturer’s protocol, and its concentration was determined using a NanoDrop 2000 spectrophotometer.

### *In vitro* experiments for ac^4^C detection

The extracted total RNA from HeLa cells was conjugated with ADB–Probe A through FTDR. The reaction was carried out at 37°C for 12 h in 50 μl mixture, including TCEP/Tris–HCl buffer (5 mM TCEP, 100 mM Tris–HCl, pH 8.8 at 25°C), 10 μg total RNA, 2 mM ADB–Probe A, and 40 U RiboLock RNase inhibitor. Upon completion of the reaction, excess Probe A was removed from the system by adsorption using Co-IRMOF-IV, a technique developed by Peng *et al.* [50]. Subsequently, the Probe A-labeled HeLa total RNA was purified using the Oligo Clean & Concentrator Kit.

The ligation reaction was carried out at 25°C for 2 h in a 10 μl reaction mixture containing 1× T4 DNA ligase buffer (40 mM Tris–HCl, 10 mM MgCl_2_, 10 mM dithiothreitol, 0.5 mM ATP, pH 7.8 at 25°C), 500 ng Probe A-labeled total RNA, 100 nM Probe B, 100 nM Probe C1, 100 nM Probe C2, 10 U RiboLock RNase inhibitor, and 5 U T4 DNA ligase. For the control group where RNA binding sites were blocked, 500 ng of Probe B-labeled HeLa total RNA was first incubated with 1 μl of 10 μM 18S-1-block or 18S-2-block at 25°C for 30 min, followed by ligation reaction.

Subsequently, 1 μl of 10 mM dNTPs, 2 μl of 10× phi29 DNA ligase buffer, 6.5 μl nuclease-free water, and 0.5 μl of 10 U/μl phi29 DNA ligase were added to the above reaction system to initiate isothermal amplification at 30°C for 4 h. Following the completion of the reaction, enzyme inactivation was achieved by incubating at 65°C for 10 min. For fluorescence detection, 30 μl of 0.5 μM MB was added to the reaction mixture, followed by incubation at 37°C in the dark for 30 min. Subsequently, fluorescent emission spectra were obtained using a PerkinElmer LS 55 spectrophotometer (PerkinElmer, USA), with excitation at 488 nm and fluorescence spectra recorded from 500 to 600 nm.

### Single-molecule inexpensive FISH

After completing all FMPLA steps, hybridization was performed using a primary probe set (final concentration of individual probes at 20 nM) and 0.5 μM secondary Cy5-labeled DNA probe in hybridization buffer (2× SSC, 10% formamide, 0.25 mg/ml RNase-free BSA, 1 mg/ml yeast tRNA, and 1 U/μl RNase inhibitor) overnight at 37°C. The cells were then washed twice with 2× SSC buffer containing 10% formamide at 37°C, followed by nuclear staining with 1 μg/ml 4',6-diamidino-2-phenylindole (DAPI).

### Selection of ac^4^C sites for analysis

Candidate ac^4^C-modified RNA sites were identified by integrating data from two independent datasets: RedaC:T-seq [32], which provides single-base resolution ac^4^C site detection, and our previously established FAM-seq [38], which offers comprehensive ac^4^C modification profiling across transcripts. By cross-referencing these datasets, we prioritized high-confidence ac^4^C sites with consistent peak calling and statistical validation.

### *In situ* imaging of ac^4^C RNA with FMPLA

#### Cell fixation and permeabilization

HeLa cells were seeded on glass-bottom dishes (20 mm) coated with poly-l-lysine hydrobromide. After the ethyl fluoroacetate treatment, the cells were fixed in prechilled solution of 4% (w/v) paraformaldehyde in PBS at room temperature for 15 min and followed by three times of washing with PBS. Subsequently, the cells were permeabilized with PBS containing 0.5% (v/v) Triton X-100 for 5 min, followed by two additional PBS washes and a final wash with 100 mM Tris–HCl (pH 7.5).

#### Orthogonal biological reaction and proximity-assisted *in situ* ligation

The fixed cells were incubated for 12 h in reaction buffer (2 mM TCEP, 100 mM Tris–HCl, 10 μg/ml salmon sperm DNA, 1 U/μl RiboLock RNase inhibitor, 0.25 mg/ml RNase-free BSA, pH 7.5 at 25°C) supplemented with 400 nM ADB–Probe A, followed by washing three times with TBST. The samples were then incubated at 37°C for 12 h in 150 μl binding buffer (2× SSC, 5 mM dithiothreitol, 1 mg/ml yeast tRNA, 1 U/μl RNase inhibitor, 10 μg/ml salmon sperm DNA, 0.25 mg/ml RNase-free BSA) supplemented with 100 nM Probe B, and subsequently washed three times with 2× SSC buffer containing 10% formamide. Then, the samples were incubated at 37°C for 3 h in 150 μl binding buffer supplemented with 100 nM Probe C1 and 100 nM Probe C2, followed by another round of washing three times with 2× SSC buffer containing 10% (v/v) formamide. Thereafter, the ligation reaction was performed by incubating the samples at 37°C for 2 h in 100 μl ligation buffer (1× T4 DNA ligase buffer, 1 U/μl T4 DNA ligase, 1 U/μl RNase inhibitor).

#### In situ RCA reaction

The RCA reaction was carried out in 100 μl amplification buffer (1× phi29 DNA polymerase reaction buffer, 0.25 mM dNTPs, 1 U/μl RNase inhibitor, 1 U/μl phi29 DNA polymerase) at 37°C for 16 h, followed by washing three times with TBST. For detection probe hybridization, the samples were incubated at 37°C for 4–6 h in 100 μl of probe hybridization buffer (2× SSC, 10 μg/ml salmon sperm DNA, 0.25 mg/ml RNase-free BSA, 1 U/μl RNase inhibitor) supplemented with 100 nM MB, followed by three washes with 2× SSC buffer containing 10% (v/v) formamide. The nuclei were then stained using 1 μg/ml DAPI, followed by three washes with 2× SSC.

#### Image acquisition

Images were captured using a Leica TCS SP8 DIVE microscope equipped with an HC PL APO CS2 63.0×/1.40 OIL objective and gadolinium hybrid (HyD) detectors, utilizing 405, 488, 552, and 638 nm laser excitation. The Leica Las X software was used for image acquisition and initial processing. The optimization of RCA conditions, imaging of the 18S rRNA 1842-ac^4^C using Probe B at varying hybridization distances, colocalization analysis, validation of FMPLA across different cell lines, and quantification of all fluorescent spots were conducted using 2D images. Other images, unless specified otherwise, were generated from maximum intensity projections of Z-stacks collected with a step size of 0.5 μm over ∼10 slices, starting below and extending above the detectable DAPI signal. Exposure times were standardized and maintained for all samples within each experiment. Representative images presented in figures are maximal intensity projections unless stated otherwise.

#### Image quantification and analysis

The FISH-quant/Big-FISH software packages were run using Python (3.6) [51]. For 3D images, the *z*-dimension was first projected to retain the maximum intensity for each *yx* pixel. Subsequently, thresholding was employed to identify and mask individual nuclei, followed by the application of the watershed algorithm to identify individual cells. The automatic spot detection method was used to determine the optimal threshold to distinguish actual spots from noise. This involved decomposing individual spots within densely bright regions by fitting Gaussian signal models to simulate as many spots as possible. The results were ultimately extracted at the cellular level to quantify FMPLA signals. Individual channel parameters were set, and all images from a single experiment were then analyzed in an automated manner and export the number of amplicons for each cell. Calculate the mean relative fluorescence intensity of cells using CellProfiler (4.2.7) [52]. Use GraphPad Prism (9.0.5) for data visualization.

### Data analysis

For imaging-based experiments, *n* represents the number of individual cells tested across a minimum of three independent biological replicates with at least three independent fields of view sampled. For all other analyses, *n* indicates the number of independent repeated experiments. When specified, *P*-values were calculated as follows: For comparisons between two conditions, a two-tailed unpaired Student's *t*-test was applied. For comparisons involving more than two conditions, a one-way ANOVA was applied. For analyses involving two variables, a two-way ANOVA followed by a Tukey post-hoc test was applied. ns, not significant; *P* < .05 (*); *P* < .01 (**); *P* < .001 (***); and *P* < .0001 (****).

## Results

### Concept and validation of FMPLA

To visualize ac^4^C in its native cellular context, as illustrated in Fig. 1, we developed FMPLA, a method based on metabolic labeling and PLA. In previous work, we confirmed that treating cells with fluoroacetate can ultimately metabolically label the presumed ac^4^C site in RNA as F-ac^4^C [38]. This process begins with the conversion of fluorine acetate to fluorine-acetyl-CoA by ACSS2, which then acts as an acetyl donor for NAT10, an acetyltransferase that transfers the fluorine-acetyl group to the N4 position of cytidine, forming F-ac^4^C. Next, Probe A, an oligo with a 5′ benzenethiol group, is labeled at the ac^4^C sites using a FTDR [53], ensuring specificity through bioorthogonal chemistry. Subsequently, Probe B, a sequence-specific RNA probe, is introduced via RISH. The spatial proximity of these probes, A and B, creates a platform for the circularization reaction, allowing Probe C1 and Probe C2, two 5′ phosphorylated DNA strands, to undergo subsequent ligation into a circular template. These strands hybridize with reserved sequences on Probes A and B, and are then ligated by T4 DNA ligase. Finally, this circular template serves as a substrate for phi29 DNA polymerase, which extends from the 3′ end of Probe A, producing rolling circle amplification products (RCPs). The resulting RCPs hybridize with signal probes (MBs), thereby generating localized fluorescent signals that can be visualized microscopically.

To validate FMPLA, its capability was first tested by detecting ac^4^C sites in 18S rRNA from HeLa cells *in vitro* (Supplementary Supplementary Fig. S1). Two well-characterized ac^4^C sites were targeted: position 1842 (∼100% stoichiometry) and position 1337 (∼80% stoichiometry) [9, 32, 54, 55]. Owing to its abundance and well-characterized ac^4^C sites, 18S rRNA served as an ideal candidate for method validation. The control group without ethyl fluoroacetate treatment displayed low fluorescence, whereas both the experimental and positive control groups targeting 18S rRNA showed strong fluorescence (Supplementary Supplementary Fig. S1A), confirming the method’s efficacy. Further analysis showed that as the hybridization distance of Probe B increased, the fluorescence intensity at positions 1842 and 1337 decreased (Supplementary Supplementary Fig. S1B and Supplementary C), suggesting that proximity is key for optimal PLA efficiency. The relationship between the probe binding site distance from the ac^4^C modification and fluorescence signal intensity was analyzed (Supplementary Supplementary Fig. S1B and Supplementary C). A statistically significant negative trend was observed, indicating that shorter distances tend to result in stronger fluorescence signals.

Control experiments provided additional evidence of FMPLA’s specificity (Supplementary Supplementary Fig. S1D and Supplementary E). Low fluorescence signals were observed when critical components, such as Probe B or Probe A, were omitted, or when Probe B targeted random sequences. Additionally, blocking the target site using a competitive strand reduced fluorescence by ∼68.1%, confirming the method’s reliance on sequence-specific binding. Similarly, omission of ethyl fluoroacetate led to a significant reduction in fluorescence, bringing it to levels comparable to those of the control group, thereby highlighting the critical role of F-ac^4^C formation in the recognition by Probe A. These results demonstrate that FMPLA achieves high sequence and site specificity *in vitro*, supporting its reliability for studying RNA modifications.

**Figure 2.**
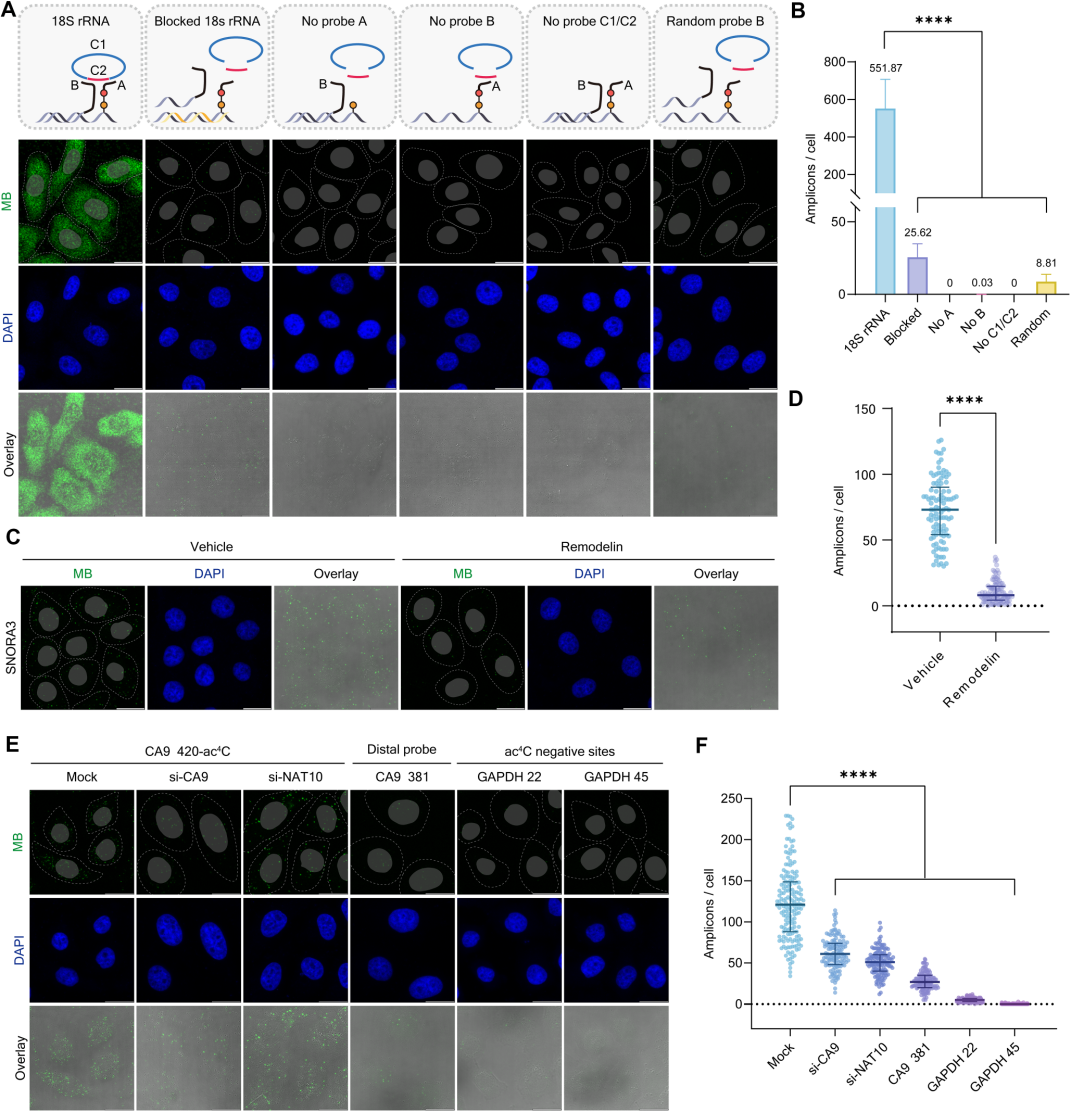
Evaluation of FMPLA for ac^4^C RNA imaging in HeLa cells. (**A**) *In situ* imaging of 18S rRNA 1842-ac^4^C in HeLa cells using FMPLA with or without the FMPLA components and various control groups. Nuclei were visualized by DAPI staining, and cell outlines are indicated by dashed lines. Scale bar: 20 μm. (**B**) Quantitative bar graph showing the number of fluorescent spots corresponding to 18S rRNA 1842-ac^4^C from the groups in (A). Values are presented as mean ± standard deviation (SD). 18S rRNA: *n* = 164; Blocked: *n* = 159; No A: *n* = 231; No B: *n* = 254; No C1/C2: *n* = 210; Random: *n* = 161. *P* < .0001 (****). (**C**) *In situ* imaging of SNORA3 RNA ac^4^C in HeLa cells with or without Remodelin treatment. Scale bar: 20 μm. (**D**) Quantification of the number of fluorescent spots in cells from the groups in (C). Scatter plots show individual data points, with solid lines representing the median with quartiles. SNORA3 vehicle: *n* = 104, mean ± SD = 72.8 ± 23.8; SNORA3 Remodelin: *n* = 104, mean ± SD = 10.6 ± 8.3. *P* < .0001 (****). (**E**) *In situ* imaging of CA9 mRNA 420-ac^4^C in HeLa cells following siRNA-mediated silencing of CA9 or NAT10. A distal probe (Probe B-CA9 381) and two negative control probes (GAPDH 22 and GAPDH 45) were included for specificity evaluation. Scale bar: 20 μm. (**F**) Quantification of the number of fluorescent spots in cells from the groups in (E). Scatter plots show individual data points, with solid lines representing the median with quartiles. Mock: *n* = 176, mean ± SD = 122.5 ± 43.5; si-CA9: *n* = 131, mean ± SD = 62.3 ± 19.5; si-NAT10: *n* = 141, mean ± SD = 50.9 ± 15.6; CA9 381: *n* = 123, mean ± SD = 27.8 ± 10.6; GAPDH 22: *n* = 130, mean ± SD = 5.09 ± 2.65; and GAPDH 45: *n* = 130, mean ± SD = 0.223 ± 0.517. *P* < .0001 (****).

To extend validation to fixed cells, FMPLA was used to visualize the 1842-ac^4^C site in 18S rRNA of HeLa cells via confocal laser scanning microscopy. Optimal RCA conditions were established (Supplementary Supplementary Fig. S2), and fluorescence imaging revealed that the 1842-ac^4^C site was predominantly localized in the cytoplasm, consistent with 18S rRNA’s role in ribosomes. A minor nuclear fluorescence signal was also observed, likely corresponding to nucleolar transcription and early processing of 18S rRNA prior to cytoplasmic export. Control experiments, similar to the *in vitro* validation, showed almost no fluorescence in groups lacking Probe A, Probe B, and Probe C1/C2, while competitive blocking reduced fluorescence by over 95.4% (Fig. 2A and B). A strong dependence on hybridization proximity was again noted (Supplementary Supplementary Fig. S3), confirming that efficient signal generation relies on close probe–target binding. Colocalization experiments further demonstrated that ac^4^C signals overlapped with 18S rRNA signals, reflecting accurate detection within subcellular compartments (Supplementary Supplementary Fig. S4). These results confirm the high specificity of FMPLA, achieved through precise probe targeting, ensuring minimal off-target effects.

Additionally, the method’s broad applicability was demonstrated across various cell lines (Supplementary Supplementary Fig. S5), including MCF-10A, HEK293T, and U2OS, underscoring its versatility across diverse cellular contexts. Using Remodelin, an effective NAT10 inhibitor [56–58], we observed a significant decrease in fluorescence spots corresponding to the ac^4^C site at position 74 of SNORA3 (small nucleolar RNA, snoRNA), indicating a substantial reduction in ac^4^C levels (Fig. 2C and D). Similarly, siRNA-mediated knockdown of CA9 and NAT10 reduced CA9 mRNA transcripts detected by FMPLA by ∼49.2% and ∼58.4% (Fig. 2E and F). These results were corroborated by reverse transcription quantitative PCR (RT-qPCR), which showed an ∼85.5% reduction in CA9 transcript levels in siRNA-treated cells (Supplementary Supplementary Fig. S6A), and western blot quantification indicating an ∼75% decrease in NAT10 protein expression (Supplementary Supplementary Fig. S6B). These results reinforce FMPLA’s target specificity toward ac^4^C-modified RNAs.

Given the ongoing debate regarding the prevalence of ac^4^C in mRNA [31], we selected RNA targets based on previously reported high-confidence ac^4^C sites identified via RedaC:T-seq [32] and FAM-seq [38]. GAPDH mRNA, which showed no evidence of ac^4^C modification, was included as a negative control to establish baseline fluorescence and assess the amplification background from non-acetylated transcripts (Fig. 2E and F). As expected, FMPLA’s detection of GAPDH yielded extremely low fluorescence signals, supporting the specificity of our target selection strategy. In parallel, we used the distal Probe B-CA9 381, which is located 39 nt upstream of the ac^4^C site at position 420 in CA9 mRNA. Compared with the site-proximal Probe B-CA9 420, the distal probe yielded ∼73.4% reduction in the number of fluorescent spots. This significant attenuation underscores the proximity requirement for efficient RCA initiation and reinforces the site-specific nature of FMPLA-based detection.

To investigate the dynamics of ac^4^C modification accumulation, FMPLA was used to analyze the CA9 420-ac^4^C site and the 18S rRNA 1842-ac^4^C site under varying ethyl fluoroacetate treatment durations. The results demonstrated a rapid accumulation of ac^4^C modifications within the first 2 h of treatment, reaching a plateau between 4 and 6 h. This was reflected in the average fluorescence intensity per cell for the 18S rRNA site (Supplementary Supplementary Fig. S7A) and the number of fluorescence puncta per cell for the CA9 mRNA site (Supplementary Supplementary Fig. S7B). These findings highlight the efficiency of FMPLA in detecting newly formed ac^4^C modifications over short time frames, providing insights into RNA modification accumulation with temporal resolution. In summary, these results highlight FMPLA’s robust capabilities for high-specificity and high-sensitivity *in situ* imaging of ac^4^C RNA at the single-cell level.

### Dynamics of RNA ac^4^C modifications across cell cycle stages

To demonstrate the broad applicability of FMPLA, the dynamics of RNA ac^4^C modifications were explored across different cell cycle stages. NAT10, the only known protein that catalyzes RNA cytosine acetylation in humans, has been reported to support mitosis by acetylating microtubule proteins, thereby promoting the stability of centrosomes and microtubules [59, 60]. However, the specific role of ac^4^C RNAs in cell cycle regulation remains inadequately understood. Investigating changes in ac^4^C modifications during mitosis offers new insights into how epigenetic transcriptomic alterations impact key cellular processes such as proliferation and division.

**Figure 3.**
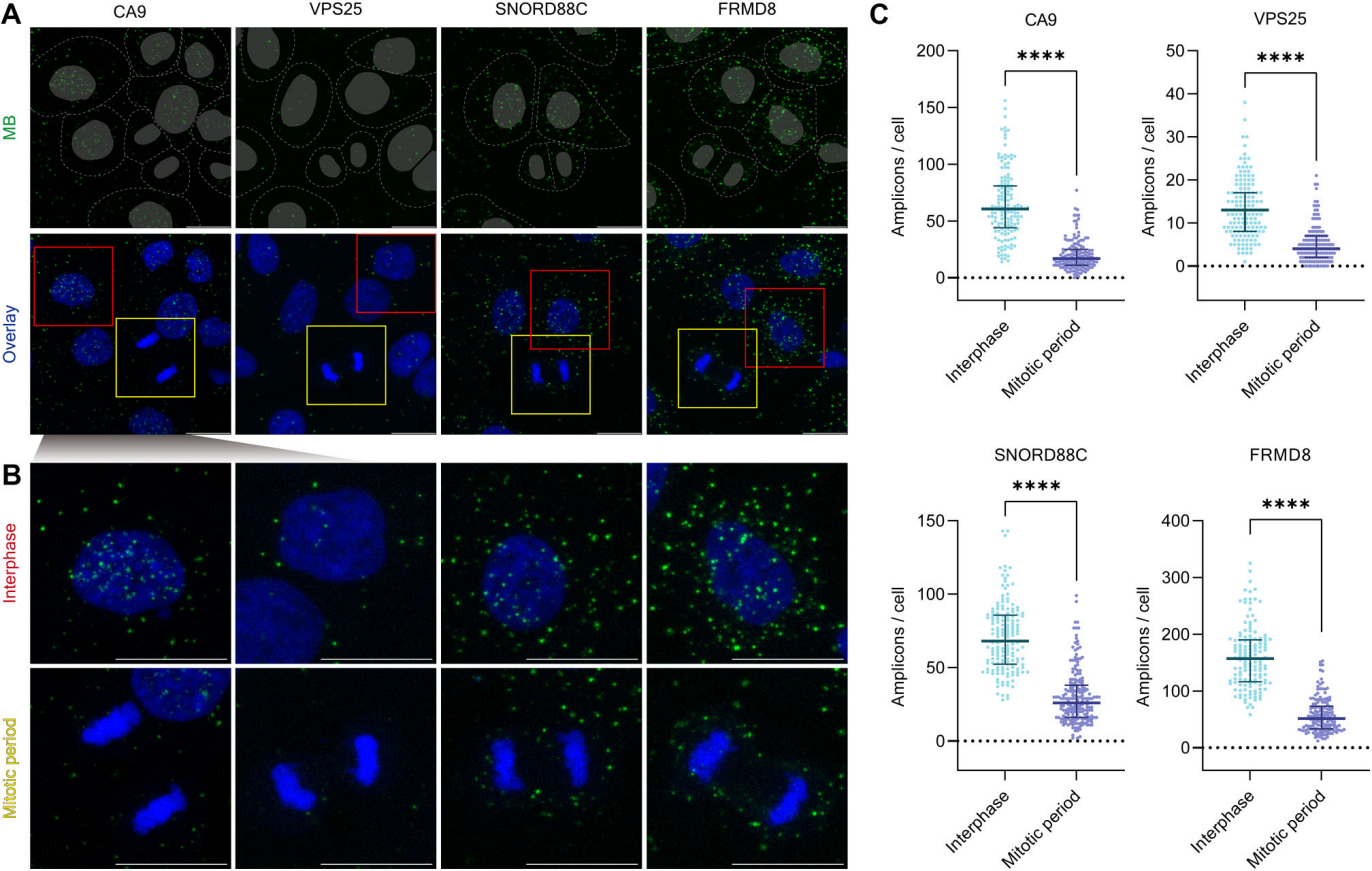
Dynamics of RNA ac^4^C modifications across cell cycle stages. (**A**) *In situ* imaging of CA9, VPS25, FRMD8, and SNORD88C RNA ac^4^C in synchronized cell cycle HeLa cells following 2 h ethyl fluoroacetate labeling using FMPLA. Nuclei were visualized by DAPI staining, and cell outlines are indicated by dashed lines. Representative mitotic and interphase cells are highlighted in yellow and red boxes, respectively. Scale bar: 20 μm. (**B**) Enlarged views of boxed regions from (A). Scale bar: 20 μm. (**C**) Quantification of the number of fluorescent spots in cells from the groups in (A). Scatter plots show individual data points, with solid lines representing the median with quartiles. CA9 interphase: *n* = 150, mean ± SD = 62.4 ± 29.7; CA9 mitotic period: *n* = 146, mean ± SD = 20.1 ± 13.5; VPS25 interphase: *n* = 149, mean ± SD = 13.3 ± 6.74; VPS25 mitotic period: *n* = 146, mean ± SD = 5.13 ± 4.23; SNORD88C interphase: *n* = 160, mean ± SD = 70.7 ± 23.2; SNORD88C mitotic period: *n* = 199, mean ± SD = 30.1 ± 18.1; FRMD8 interphase: *n* = 141, mean ± SD = 159.6 ± 54.6; and FRMD8 mitotic period: *n* = 150, mean ± SD = 57.5 ± 30.7. *P* < .0001 (****).

To investigate the dynamic regulation of ac^4^C modifications across different cell cycle states, we employed a double thymidine block and release to synchronize cells in the S phase, thereby generating a more uniform population as they entered mitosis [49]. This synchronization facilitated temporal resolution in detecting dynamic changes in ac^4^C modifications during mitotic progression. To this end, we performed ethyl fluoroacetate metabolic labeling either 2 h (Fig. 3A and B, and Supplementary Supplementary Figs S8 and Supplementary S9) or 8 h (Supplementary Supplementary Fig. S9) before cell fixation, during the second thymidine release. Then, FMPLA was utilized to visualize ac^4^C-modified RNAs—namely CA9 (mRNA), VPS25 (mRNA), FRMD8 (miscRNA), and SNORD88C (snoRNA).

While distribution patterns varied among the different ac^4^C RNAs, the trend in their quantities across various cell cycle stages remained consistent. First, interphase cells exhibited the highest number of fluorescent spots (Fig. 3C and Supplementary Supplementary Fig. S9). Second, the average number of fluorescent spots in mitotic cells was reduced by ∼56%–77% compared with interphase cells (Fig. 3C). Notably, the ac^4^C modification levels in mitotic cells showed greater cellular heterogeneity, as reflected in their larger coefficient of variation. Finally, anaphase and telophase cells generally exhibited the fewest number of fluorescent spots, suggesting a decline in ac^4^C modifications as cells progress through these later mitotic stages. Importantly, both 2- and 8-h ethyl fluoroacetate labeling conditions produced consistent trends across the cell cycle, with a greater number of fluorescent spots observed in the 8-h group (Supplementary Supplementary Fig. S9), as expected.

The data suggest that ac^4^C modifications are more abundant during earlier stages and decrease as cells progress toward the later stages of mitosis, possibly indicating dynamic regulation. Fluctuations in ac^4^C levels during metaphase may signify a transitional state, while the decline observed in later stages could reflect selective removal or active degradation of ac^4^C-modified RNAs, suggesting a role in modulating gene expression throughout mitosis [61–63]. Importantly, the acetylation dynamics observed during the cell cycle may yield new insights into the regulation and functional implications of differential mRNA acetylation levels in future investigations. However, further experiments are required to directly assess these potential mechanisms. In addition, the successful implementation of the 2-h pulse labeling not only underscores FMPLA’s high sensitivity and temporal resolution, but also provides a robust technical foundation for investigating the rapid dynamics of ac^4^C modifications, stress-induced epitranscriptomic responses, transient RNA modifications, and applications in primary or sensitive cell systems.

### Cisplatin-induced changes in ac^4^C RNA modifications

To demonstrate the versatility of FMPLA in various contexts, the relationship between changes in ac^4^C modification and drug action was investigated. Cisplatin, a widely used chemotherapeutic agent, binds to DNA, disrupts DNA repair mechanisms, and inhibits cancer cell proliferation [64, 65]. However, resistance to cisplatin poses a significant clinical challenge. Recent studies have indicated that cisplatin activates the NF-κB signaling pathway, which upregulates NAT10 transcription [19, 25, 66, 67]. The accumulation of NAT10 in the nucleus leads to increased ac^4^C modifications in RNAs, thereby enhancing DNA repair and promoting cisplatin resistance [22, 68–70].

**Figure 4.**
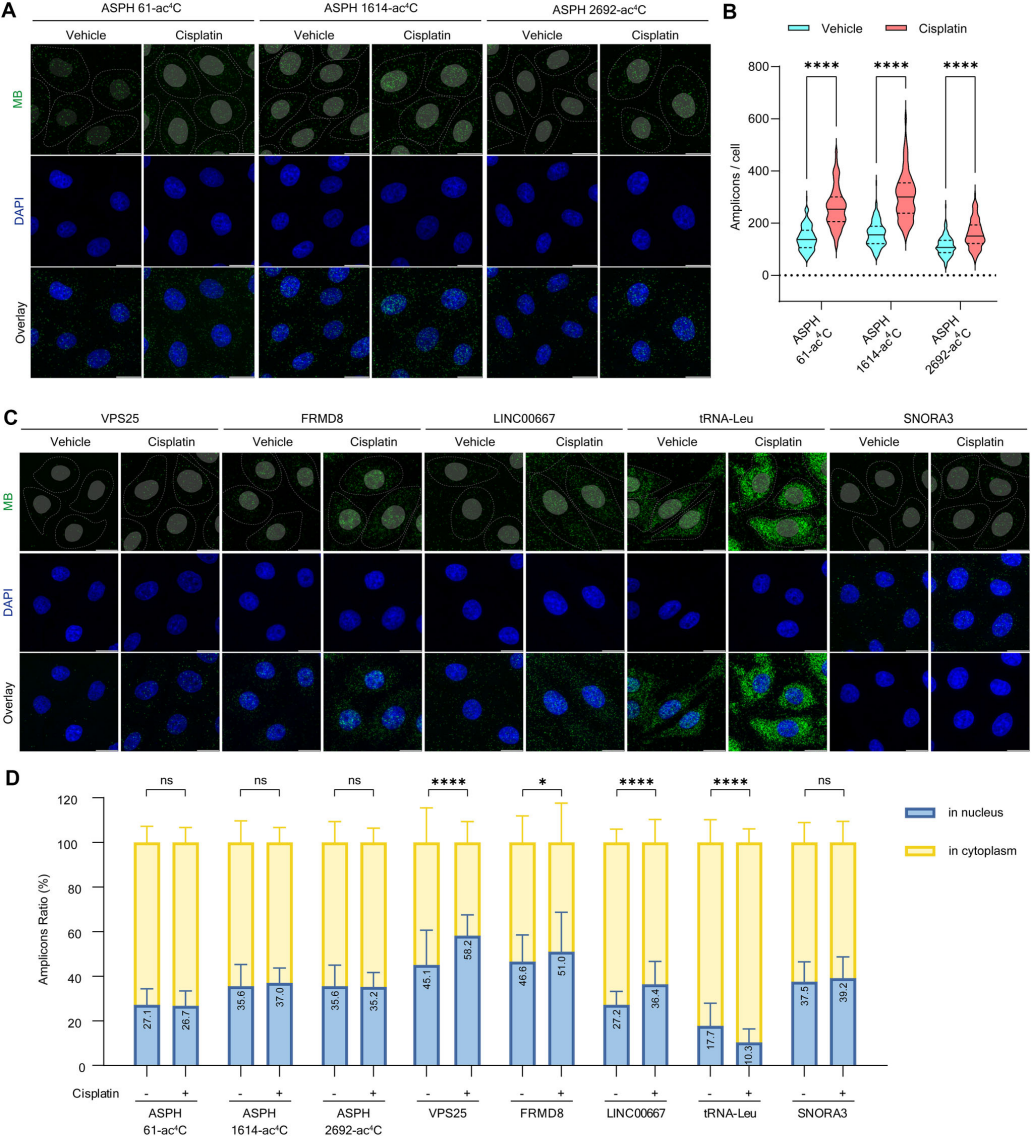
Visualization of the changes in ac^4^C modification following cisplatin treatment. (**A**) *In situ* imaging of ASPH mRNA 61-ac^4^C, 1614-ac^4^C, and 2692-ac^4^C in HeLa cells treated with or without cisplatin using FMPLA. Nuclei were visualized by DAPI staining, and cell outlines are indicated by dashed lines. Scale bar: 20 μm. (**B**) Violin plot showing the number of fluorescent spots in cells from the groups in (A). Solid lines represent medians, while dashed lines indicate quartiles. ASPH-61 vehicle: *n* = 167, mean ± SD = 142.3 ± 45.6; ASPH-1614 vehicle: *n* = 165, mean ± SD = 158.0 ± 47.5; ASPH-2692 vehicle: *n* = 159, mean ± SD = 111.6 ± 36.9; ASPH-61 cisplatin: *n* = 134, mean ± SD = 261.4 ± 77.6; ASPH-1614 cisplatin: *n* = 132, mean ± SD = 305.6 ± 91.6; and ASPH-2692 cisplatin: *n* = 126, mean ± SD = 159.2 ± 54.2. *P* < .0001 (****). (**C**) *In situ* imaging of VPS25, FRMD8, LINC00667, tRNA-Leu, and SNORA3 RNA ac^4^C in HeLa cells treated with or without cisplatin using FMPLA. Scale bar: 20 μm. (**D**) Stacked bar chart showing the distribution of fluorescent RNA ac^4^C spots in the nucleus and cytoplasm of HeLa cells for ASPH (61-, 1614-, and 2692-ac^4^C), VPS25, FRMD8, LINC00667, tRNA-Leu, and SNORA3, with or without cisplatin treatment. Values are presented as mean ± SD. ASPH-61 vehicle: *n* = 167; ASPH-1614 vehicle: *n* = 165; ASPH-2692 vehicle: *n* = 159; ASPH-61 cisplatin: *n* = 134; ASPH-1614 cisplatin: *n* = 132; ASPH-2692 cisplatin: *n* = 126. VPS25 vehicle: *n* = 199; VPS25 cisplatin: *n* = 128; FRMD8 vehicle: *n* = 191; FRMD8 cisplatin: *n* = 142; LINC00667 vehicle: *n* = 122; LINC00667 cisplatin: *n* = 115; tRNA-Leu vehicle: *n* = 195; tRNA-Leu cisplatin: *n* = 98; SNORA3 vehicle: *n* = 172; and SNORA3 cisplatin: *n* = 164. ns, not significant; *P* < .05 (*); *P* < .0001 (****).

To explore the impact of cisplatin on ac^4^C modifications in both coding and non-coding RNAs, the FMPLA technique was employed. In HeLa cells, the modification levels of ac^4^C in ASPH mRNA were initially assessed. Cisplatin treatment resulted in a significant increase in ac^4^C modifications within the coding sequence region, ∼93.4%, followed by an increase of ∼83.7% in the 5′ UTR, and the least increase observed in the 3′ UTR at around 42.7% (Fig. 4A and B). This increase in modification levels was particularly pronounced for the VPS25 mRNA at the 324-ac^4^C site, where the number of fluorescent spots increased by ∼802.7% (Supplementary Supplementary Fig. S10). Notably, while cisplatin influenced the overall level of ac^4^C modifications in ASPH mRNA, it did not significantly alter its intracellular localization. When applying FMPLA to the ncRNAs LINC00667 (long non-coding RNA, lncRNA) and FRMD8, a notable approximately two-fold increase in the number of green fluorescent spots was observed, along with an accumulation of these fluorescent spots in the nucleus (Fig. 4C and D, and Supplementary Supplementary Fig. S10). This suggests that cisplatin not only enhances ac^4^C modification levels in ncRNA but also significantly influences their subcellular localization, potentially contributing to their stability and function during DNA repair processes [71]. Additionally, an upregulation of ac^4^C levels on tRNA-Leu was also observed, alongside a preferential localization in the cytoplasm. The cytoplasmic localization of tRNA-Leu highlights dynamic cellular responses to stress, likely tied to enhanced translation. These findings underscore the powerful utility of FMPLA in visualizing the dynamics of ac^4^C responses to cisplatin and suggest its potential in investigating mechanisms of drug resistance.

### Visualization of RNA ac^4^C modulation in DOX-induced drug-resistant cells

To demonstrate the applicability of FMPLA in drug research, we explored its use in visualizing ac^4^C RNA in the context of drug resistance in CML. CML is a malignant hematopoietic disorder originating from stem cells, accounting for over 15% of adult leukemia cases [72, 73]. The widely used chemotherapy agent DOX has been shown to effectively inhibit the progression of various cancers, including CML [74, 75]. However, the emergence of multidrug resistance in cancer cells significantly limits the efficacy of DOX [76, 77]. Recent studies have underscored the importance of RNA modifications, such as ac^4^C, in cancer progression, development, and drug response [78]. Changes in ac^4^C levels can influence RNA stability and translation efficiency, thereby affecting cellular sensitivity to drugs. These modifications could serve as both biomarkers of resistance and potential therapeutic targets, making systematic investigation of ac^4^C changes in response to drug treatment essential for understanding resistance mechanisms.

**Figure 5.**
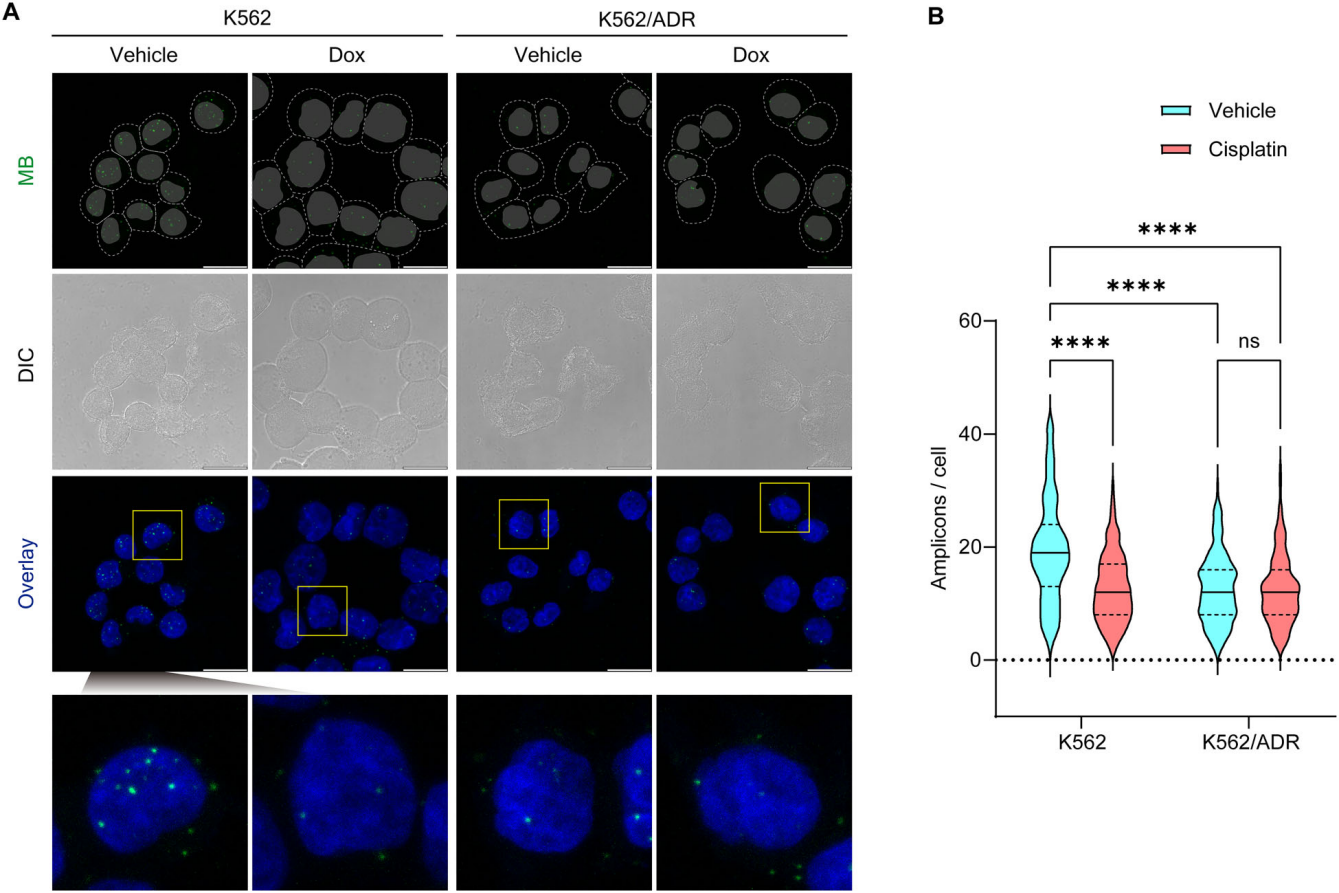
Visualization of RNA ac^4^C modulation in DOX-induced drug-resistant cells. (**A**) *In situ* imaging of TOMM7 RNA ac^4^C in K562 and K562/ADR cells treated with or without DOX using FMPLA. Nuclei were visualized by DAPI staining, and cell outlines are indicated by dashed lines. Scale bar: 20 μm. (**B**) Violin plot showing the number of fluorescent spots in cells from the groups in (A). Solid lines represent medians, and dashed lines indicate quartiles. K562 vehicle: *n* = 253, mean ± SD = 19.3 ± 9.1; K562 DOX: *n* = 194, mean ± SD = 12.7 ± 6.1; K562/ADR vehicle: *n* = 249, mean ± SD = 12.7 ± 6.2; K562/ADR DOX: *n* = 190, mean ± SD = 12.5 ± 6.1. ns, not significant; *P* < .0001 (****).

K562 cells and their DOX-resistant counterpart, K562/ADR cells, were utilized to visualize ac^4^C RNA before and after DOX treatment using FMPLA. As shown in Fig. 5, a significant reduction in the number of fluorescent spots corresponding to TOMM7 mRNA was observed in both K562/ADR and DOX-treated K562 cells compared to untreated K562 cells (Fig. 5B), indicating a marked decrease in ac^4^C modification levels under these conditions. Notably, no significant difference was found between DOX-treated and untreated K562/ADR cells, indicating that ac^4^C levels in these resistant cells remain stable regardless of drug exposure. This stability suggests that K562/ADR cells may possess regulatory mechanisms that preserve ac^4^C levels, potentially contributing to their resistance to DOX. Understanding these differential responses is critical for unraveling the mechanisms underlying drug resistance, paving the way for more effective therapeutic strategies. This finding underscores the potential of FMPLA to explore the molecular mechanisms of drug sensitivity and resistance, informing future cancer therapies [79, 80].

## Discussion

In this study, we developed and validated FMPLA, an innovative method for the *in situ* detection and visualization of ac^4^C RNA modifications. Given the emerging role of ac^4^C in regulating RNA function and its potential links to diverse disease mechanisms, developing highly sensitive and specific tools for mapping these modifications is crucial. By integrating metabolic labeling with a PLA, FMPLA offers high specificity and sensitivity across a wide array of RNA species, including rRNA, mRNA, tRNA, snoRNA, and lncRNA. The method utilizes a fluoroacetic acid derivative to label new ac^4^C sites as F-ac^4^C, followed by bioorthogonal chemistry, facilitating precise visualization of ac^4^C-modified RNAs within cellular environments. Its versatility across various cell lines and RNA types underscores its broad applicability for studying RNA modifications.

The key advantages of the FMPLA method include the following: (i) a discrete and robust fluorescence signal derived from RCA, enabling high-sensitivity spatial analysis of ac^4^C-modified RNA; (ii) avoidance of the limitations inherent in antibody-based methods, such as instability, through the use of fluoroacetate metabolic labeling, rendering it a simpler, more efficient, and cost-effective approach; (iii) high specificity, achieved through site- and sequence-specific probes, ensuring accurate *in situ* detection of ac^4^C-modified RNA while minimizing nonspecific signals; and (iv) flexibility in probe design, allowing for customization to target any ac^4^C-modified RNA molecule.

FMPLA offers significant advantages by providing valuable spatial resolution information of RNA modifications at the cellular level, a capability that sequencing alone lacks. Furthermore, FMPLA enables orthogonal validation of specific RNA ac^4^C sites identified in sequencing studies, thereby enhancing the reliability of the findings. Compared to traditional *in situ* hybridization, which remains an effective tool for assessing RNA abundance and localization, FMPLA captures modification-specific features. In the future, the integration of these complementary imaging tools may provide deeper insights, such as the proportion of ac^4^C modifications at specific sites, leading to a more comprehensive understanding of RNA and its modifications. Moreover, FMPLA uniquely enables visualization of newly formed ac^4^C modifications, which are rapidly generated following the introduction of the metabolic labeling reagent. In contrast to traditional antibody-based methods, which detect the total pool of ac^4^C-modified RNAs, FMPLA can provide a snapshot of the most recent modifications. This advantage enables the study of dynamic ac^4^C incorporation in real time, particularly during rapid cellular processes such as stress responses, gene expression changes, or cell cycle transitions. By focusing on nascent ac^4^C-modified RNA, FMPLA enables researchers to investigate how these modifications actively contribute to biological processes as they unfold.

However, this focus on newly synthesized modifications also presents a limitation. FMPLA primarily captures RNA species undergoing modification at the time of labeling, potentially overlooking older ac^4^C modifications that may still play crucial roles in cellular function. Additionally, incorporating fluorine into RNA might alter its natural conformation, potentially affecting downstream biological processes [81–85]. Although our study did not reveal significant issues, addressing these potential concerns is crucial for enhancing the precision and consistency of future applications.

In conclusion, the FMPLA method has proven invaluable in elucidating critical insights into RNA regulation, particularly regarding cell cycle-dependent dynamics and differential responses to chemotherapeutic agents in cancer models. These findings advance our understanding of the roles that RNA modifications play in cellular processes and highlight FMPLA’s potential to inform the development of innovative therapeutic strategies in oncology. Future research should focus on expanding the method’s applications and refining its capabilities to fully harness its potential in diverse biological and clinical contexts.

## Supplementary Material

gkaf464_Supplemental_File

## Data Availability

Data supporting the findings of this study are available upon request due to the size and number of imaging datasets.
