# Correction to ‘Single-cell imaging of *N*^4^-acetylcytidine-modified RNA using fluorine metabolic labeling mediated proximity ligation assay’

**DOI:** 10.1093/nar/gkag010

**Published:** 2026-01-14

This is a correction to: Qi Wang, Yuhao Du, Shen Yan, Ziang Lu, Yongling Tang, Feng Xiao, Fuling Zhou, Xiang Zhou, Single-cell imaging of N4-acetylcytidine-modified RNA using fluorine metabolic labeling mediated proximity ligation assay, Nucleic Acids Research, Volume 53, Issue 10, 10 June 2025, gkaf464, https://doi.org/10.1093/nar/gkaf464

The following changes have been made to the originally published manuscript.

In Figure 1, “sodium fluoroacetate” has been corrected to “ethyl fluoroacetate”.

In **Materials and methods section**, under heading ‘*In situ* imaging of ac4C RNA with FMPLA **Cell fixation and permeabilization’**, the second sentence of the paragraph is emended to read: “After the ethyl fluoroacetate treatment,[…]” instead of: “After the sodium fluoroacetate treatment,[…]”.

These changes do not affect the results, discussion and conclusions presented in the article.

