# Supplementary material for: Single-cell imaging of N4-acetylcytidine-modified RNA using fluorine metabolic labeling mediated proximity ligation assay
Source: Nucleic Acids Res. 2025 Jun 4;53(10):gkaf464. doi: 10.1093/nar/gkaf464 (PMC12135178; doi:10.1093/nar/gkaf464)
Supplement: gkaf464_Supplemental_File [file gkaf464_supplemental_file.pdf]

SUPPLEMENTARY INFORMATION

Single-cell imaging of N^4-acetylcytidine modified RNA using fluorine metabolic labeling mediated proximity ligation assay

Qi Wang[1,†], Yuhao Du[2,†], Shen Yan[2], Ziang Lu[2], Yongling Tang[2], Feng Xiao[2,*], Fuling Zhou[1,*], Xiang Zhou[2,*]

[1] Department of Hematology of Zhongnan Hospital, Taikang Center for Life and Medical Sciences, Wuhan University, Wuhan, 430071, China

[2] College of Chemistry and Molecular Sciences, Wuhan University, Wuhan, Hubei, 430072, China

[†] Joint Authors

[*] To whom correspondence should be addressed. Email: xzhou@whu.edu.cn.

Correspondence may also be addressed to: xiaofeng_97@whu.edu.cn, zhoufuling@whu.edu.cn.

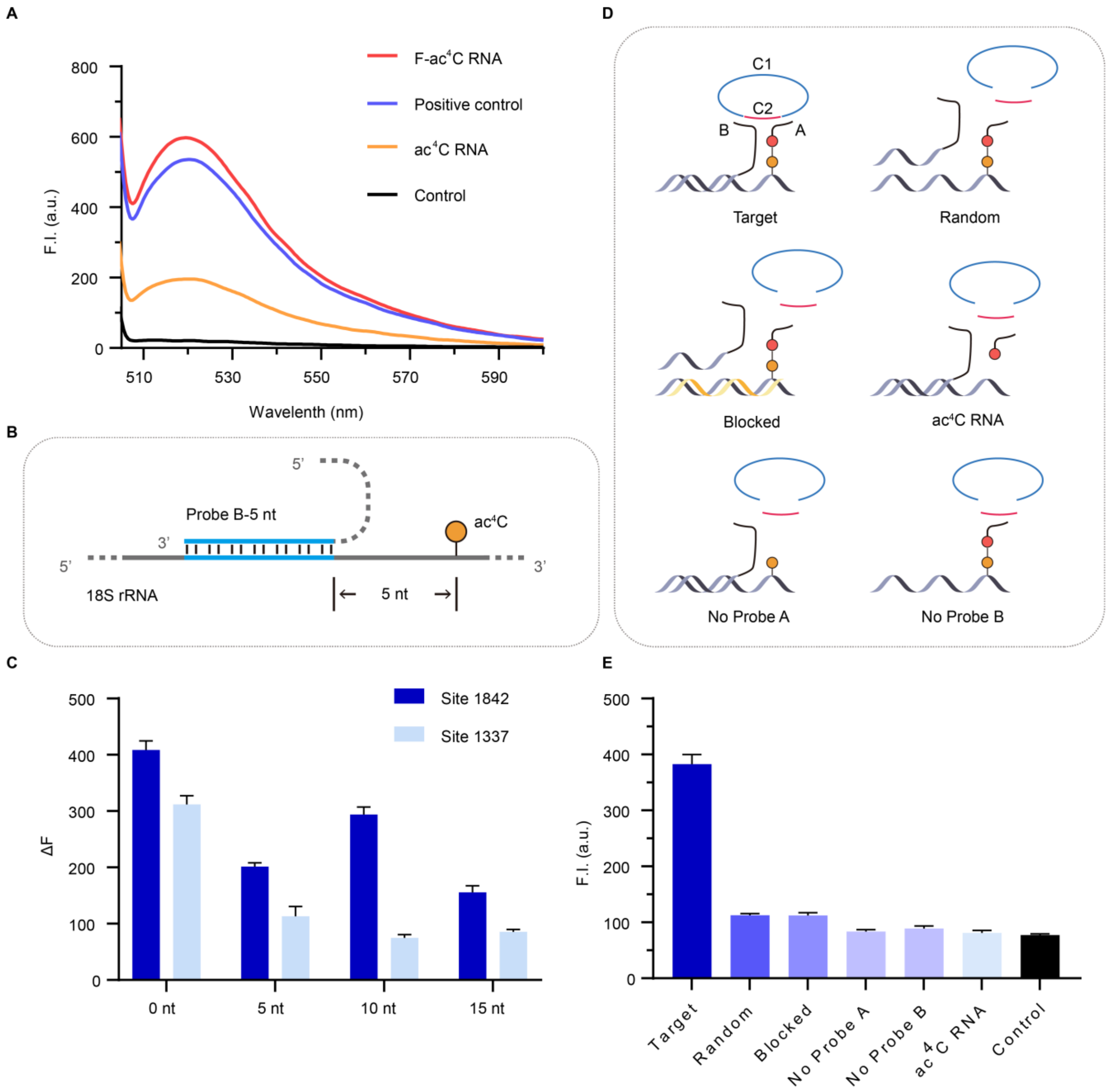

Figure S1. In vitro validation of FMPLA.

(A) Fluorescence emission spectra of rolling circle amplification (RCA) products detected using FMPLA. The "ac^{4}C RNA" group represents samples from cells not treated with fluoroacetate ethyl ester. The "Positive Control" group includes samples processed with Probe C-Positive-1 and Probe C-Positive-2 to ensure the formation of circular templates. The "Control" group consists of samples without added RNA. (B) Schematic illustration of Probe B binding sites on 18S rRNA. A series of Probe B was designed to hybridize at upstream positions 0 nt, 5 nt, 10 nt, and 15 nt from the ac^{4}C-modified sites at positions 1842 and 1337 on 18S rRNA. (C) Bar graph showing the fluorescence intensity at 520 nm for FMPLA products corresponding to the eight Probe B variants mentioned in (B) and their respective control groups. Values are presented as mean ± SD; n = 3. Linear trend analysis and one-way ANOVA were performed to evaluate the relationship between probe binding site distance and fluorescence signal intensity. A significant linear trend was observed ($P < 0.0001$ for both site 1842 and site 1337), with shorter distances associated with stronger signals. The linear component explained 58.66% of the variance for site 1842 and 68.24% for site 1337 ($R^2 = 0.5866$ and $R^2 = 0.6824$), while

additional nonlinear effects explained 40.22% and 30.7% of the variance for site 1842 and site 1337, respectively (R^2_{change} = 0.4068 and R^2_{change} = 0.3103). (D) Schematic representation of FMPLA components and control groups. (E) Bar graph showing the fluorescence intensity at 520 nm for RCA products and their respective control groups, as described in (D). Values are presented as mean ± SD; n = 3.

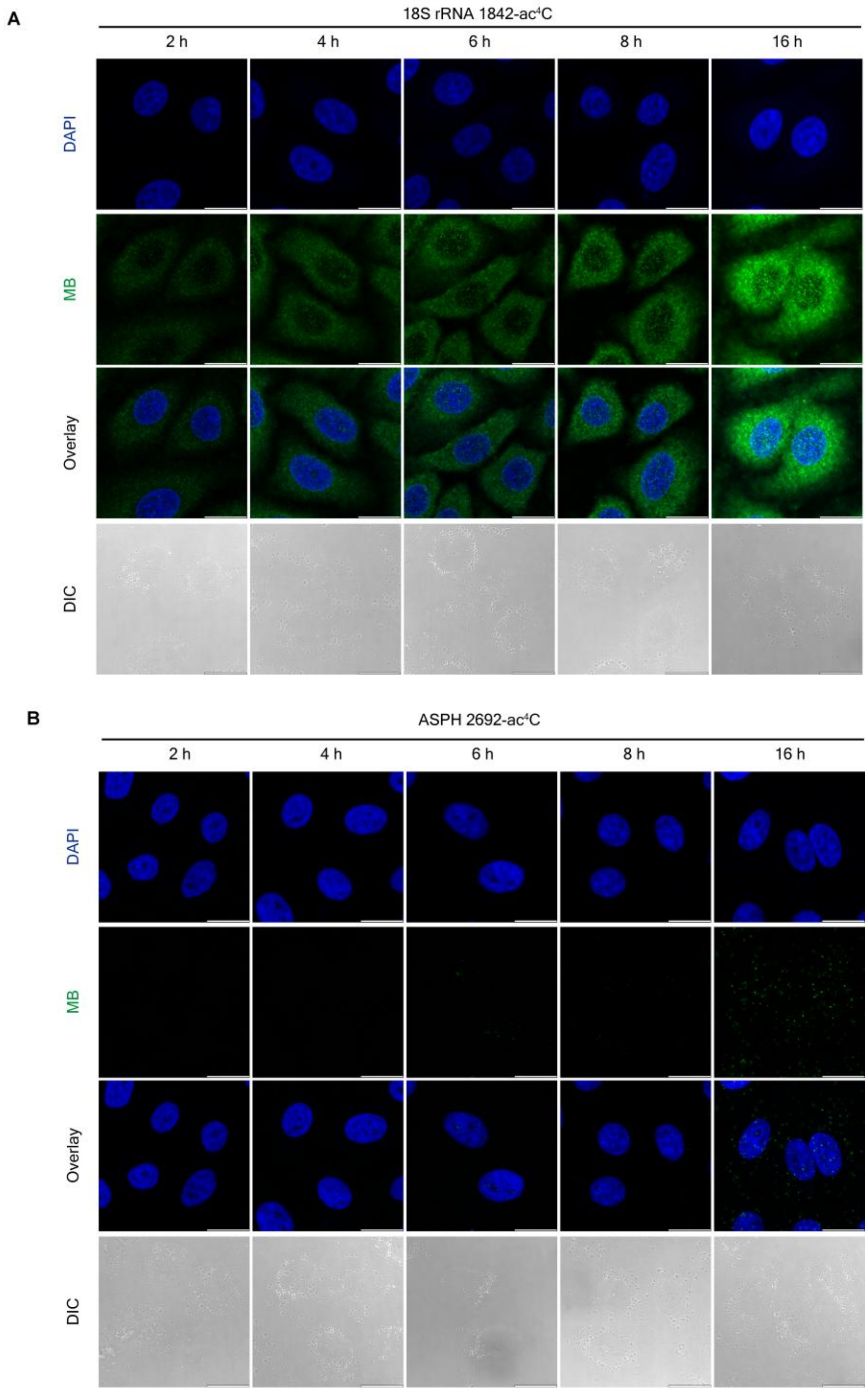

Figure S2. Optimization of RCA time conditions for FMPLA.

FMPLA was utilized for in situ imaging of 18S rRNA 1842-ac^{4}C (A) and ASPH 2692-ac^{4}C (B) in HeLa cells, employing rolling circle amplification reaction times of 2, 4, 6, 8, and 16 h. The optimal RCA duration was determined to be 16 h. Bright green dots represent amplicons of RNA ac^{4}C, the cell nuclei are shown in blue (DAPI). Scale bar: 20 μm.

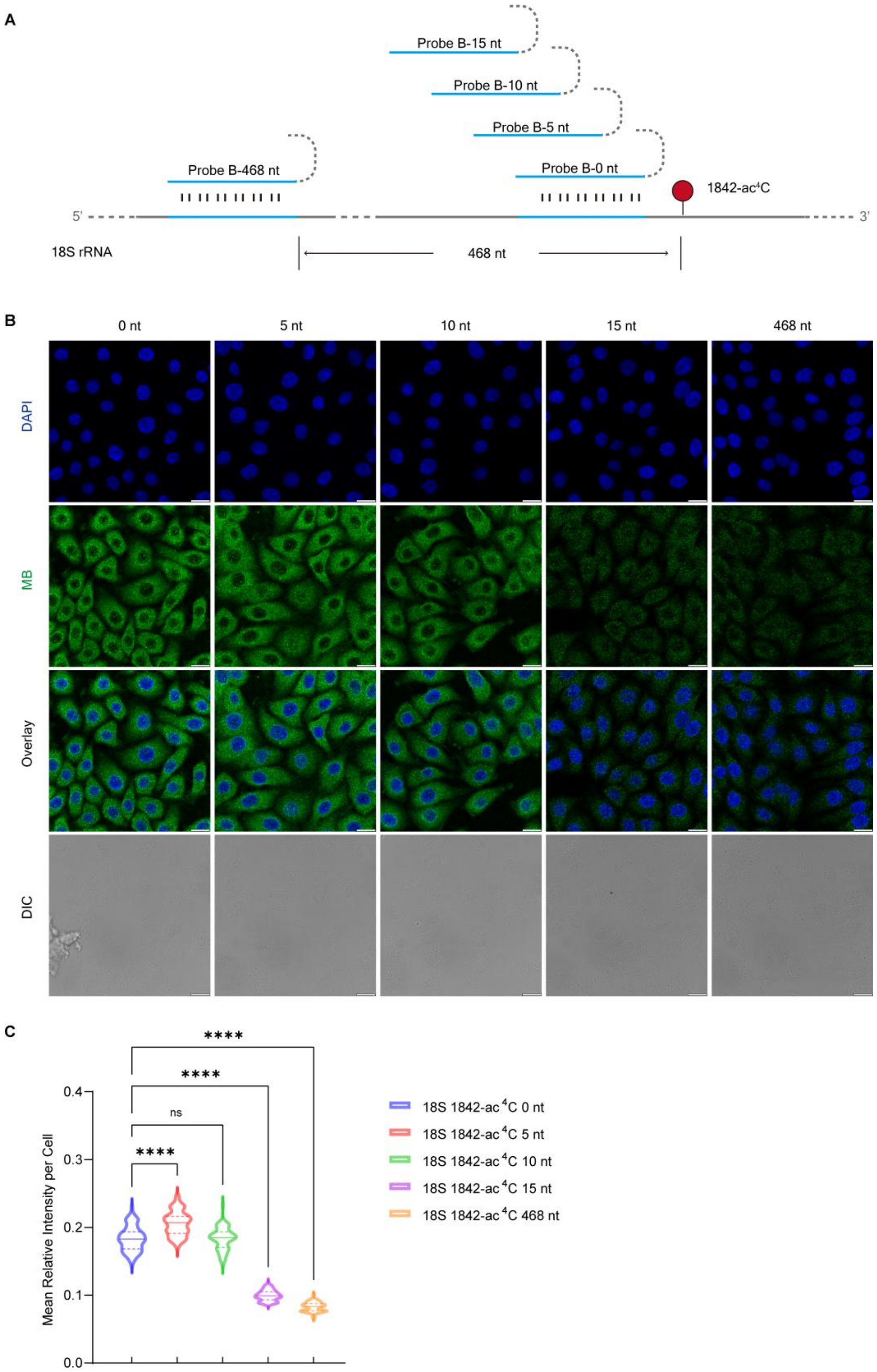

Figure S3. Probe B binding and FMPLA imaging of 18S rRNA 1842-ac^{4}C.

(A) Schematic representation of the binding sites of various Probe B on 18S rRNA. (B) FMPLA imaging of 18S rRNA 1337-ac^{4}C and 1842-ac^{4}C in HeLa cells, using Probe B hybridizing at distances of 0 nt, 5 nt, 10 nt, 15 nt, and 468 nt from 18S rRNA 1842-ac^{4}C, and Probe B hybridizing at 0 nt from 18S rRNA 1337-ac^{4}C. Bright green dots represent amplicons of RNA ac^{4}C, the cell nuclei are shown

in blue (DAPI). Scale bar: 20 µm. (C) Violin plot quantifying the average relative fluorescence intensity detected by FMPLA in the groups shown in (B). The solid line represents the median, and dashed lines indicate the quartiles. 18S-1842-0 nt: n = 93, mean ± SD = 0.1833 ± 0.0181; 18S-1842-5 nt: n = 70, mean ± SD = 0.2059 ± 0.0178; 18S-1842-10 nt: n = 81, mean ± SD = 0.1832 ± 0.0165; 18S-1842-15 nt: n = 75, mean ± SD = 0.0995 ± 0.0079; 18S-1842-468 nt: n = 77, mean ± SD = 0.0829 ± 0.0067. ns, not significant; $P < 0.0001$ (****).

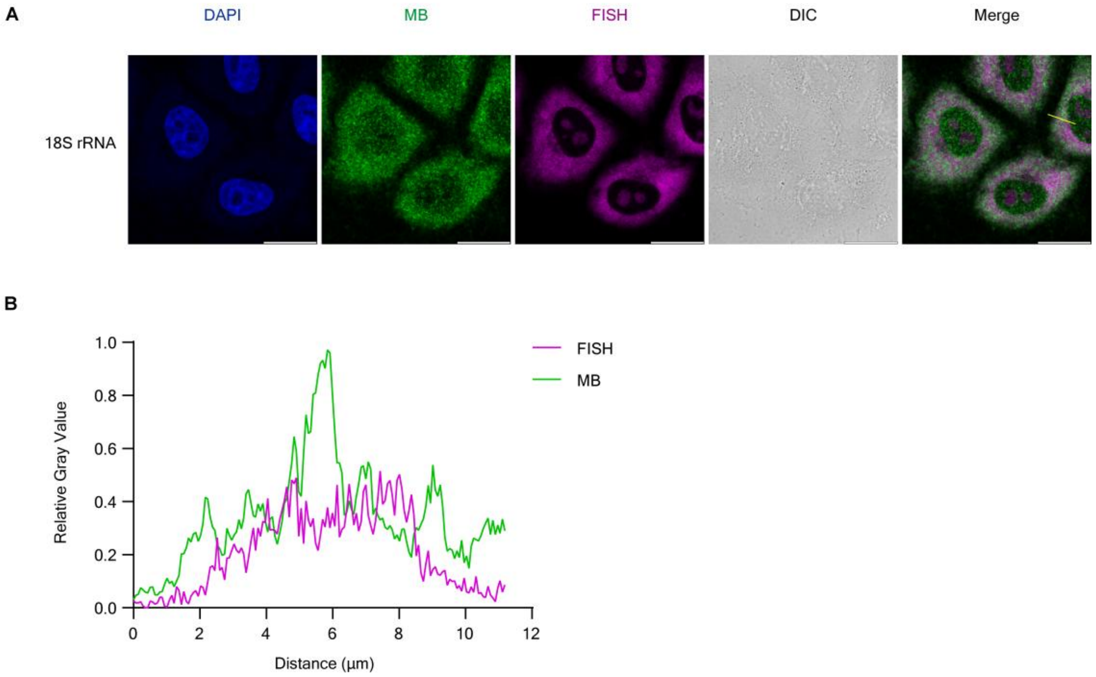

Figure S4. Co-localization analysis of FMPLA and smiFISH.

(A) CLSM images of 18S rRNA 1842-ac^{4}C visualized using FMPLA and 18S rRNA visualized using smFISH probes. Bright green dots represent amplicons of 18S rRNA 1842-ac^{4}C, while 18S rRNA is shown in magenta (Cy5). (B) Colocalization analysis between 18S rRNA 1842-ac^{4}C detected by FMPLA and 18S rRNA detected by smiFISH. The Manders' colocalization coefficient values (M_1 = 0.963, M_2 = 0.97) demonstrated a significant colocalization between the two fluorescent signals. M_1 and M_2 represent the fraction of colocalized signals between and MB the FISH probe relative to the total signal of either 18S rRNA 1842-ac^{4}C or 18S rRNA.

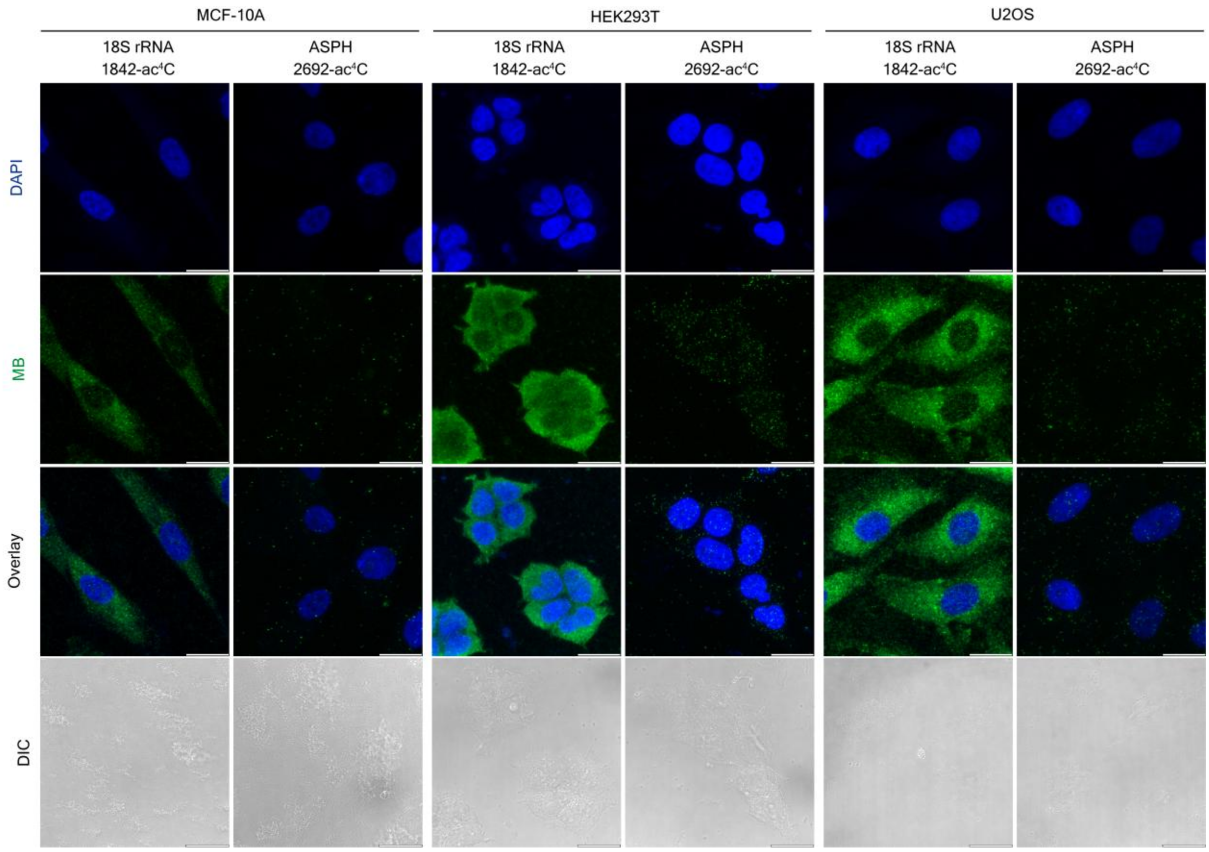

Figure S5. Validation of FMPLA versatility across different cell lines.

FMPLA imaging of 18S rRNA 1842-ac^{4}C and ASPH 2692-ac^{4}C was performed in MCF-10A, HEK239T, and U2OS cells. Bright green dots represent amplicons of RNA ac^{4}C, the cell nuclei are shown in blue (DAPI). Scale bar: 20 μm.

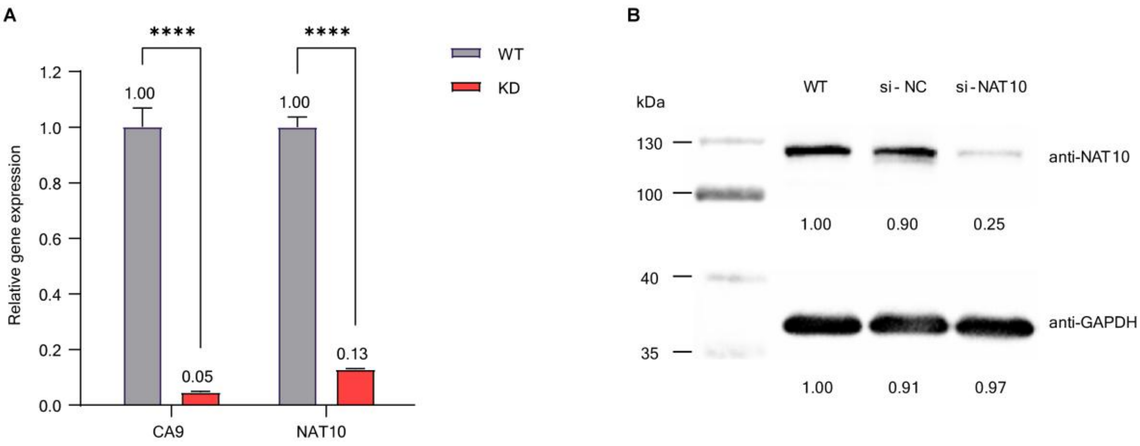

Figure S6. Validation of siRNA-Mediated Knockdown Efficiency of CA9 and NAT10 mRNA.

(A) Bar graph showing the relative expression levels of CA9 and NAT10 mRNAs. Total RNA was extracted from HeLa cells treated with siRNA targeting CA9 (siRNA-CA9) and NAT10 (siRNA-NAT10), followed by RT-qPCR analysis. GAPDH was used as the internal reference control. Values are presented as mean ± SD (n = 3). $P < 0.0001$ (****). (B) Protein levels of NAT10 in Hela cells transfected with siRNA-NAT10 and negative control siRNA (siRNA-NC) were analyzed by western blot. GAPDH was included as an internal reference control.

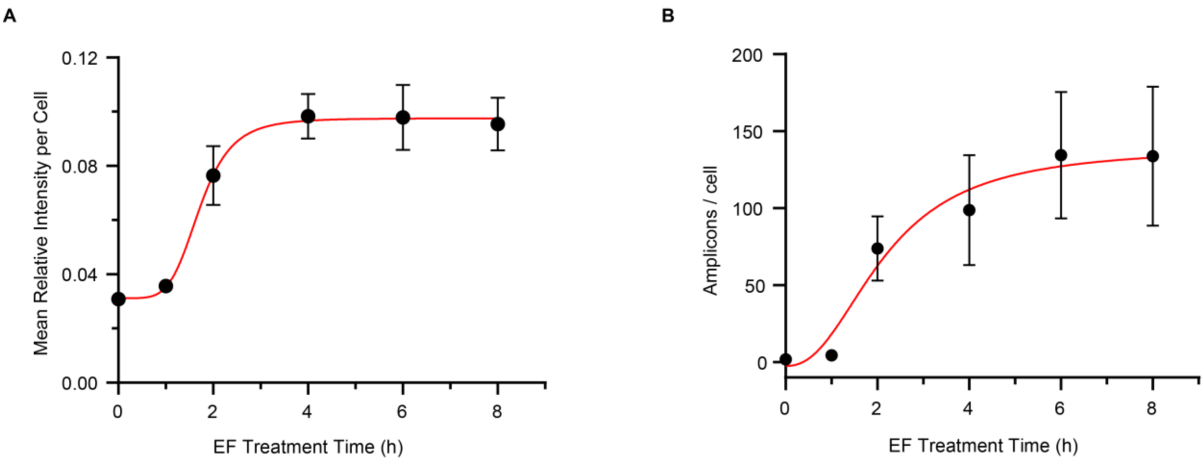

Figure S7. Time-dependent accumulation of ac^{4}C modifications

HeLa cells were treated with ethyl fluoroacetate for 0, 1, 2, 4, 6, and 8 h to assess labeling efficiency of ac^{4}C modifications via FMPLA. (A) Mean relative fluorescence intensity per cell measured for 18S rRNA 1842-ac^{4}C, showing rapid accumulation of fluorescence signal and a plateau reached at approximately 4 h. Values are presented as mean ± SD (n > 100). The curve was fitted using a 4-parameter logistic (4PL) model (red line), with IC_{50} = 1.716 h, HillSlope = 5.048, and R^2 = 0.9193. (B) Amplicons per cell detected for the CA9 420-ac^{4}C, illustrating a similar trend of rapid accumulation with a plateau observed at 6 h. Values are presented as mean ± SD (n > 100). The curve was fitted using a 4-parameter logistic (4PL) model (red line), with IC50 = 2.152 h, HillSlope = 2.311, and R^2= 0.7218.

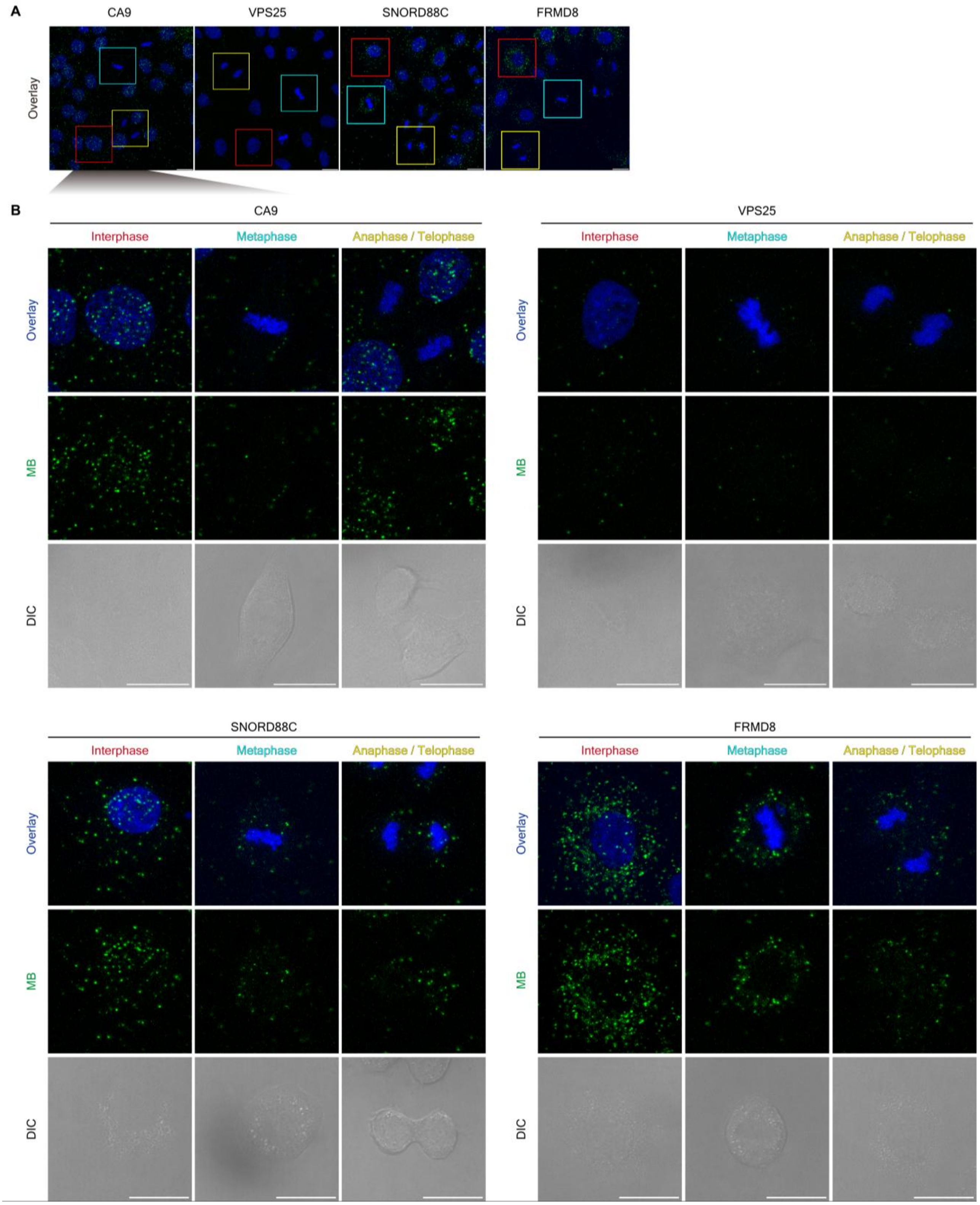

Figure S8. In situ FMPLA imaging of ac^4C-modified RNAs in HeLa cells across cell cycle phases.

(A) In situ FMPLA imaging of ac^4C-modified CA9, VPS25, FRMD8, and SNORD88C RNA in HeLa cells after cell cycle synchronization and 2 h ethyl fluoroacetate labeling. Bright green dots represent RNA ac^4C amplicons. Representative interphase cells are marked with red boxes, metaphase cells with cyan boxes, and anaphase/telophase cells with yellow boxes. Scale bar: 20 µm. (B) Enlarged views of the cells within the red, cyan, and yellow boxes from (A).

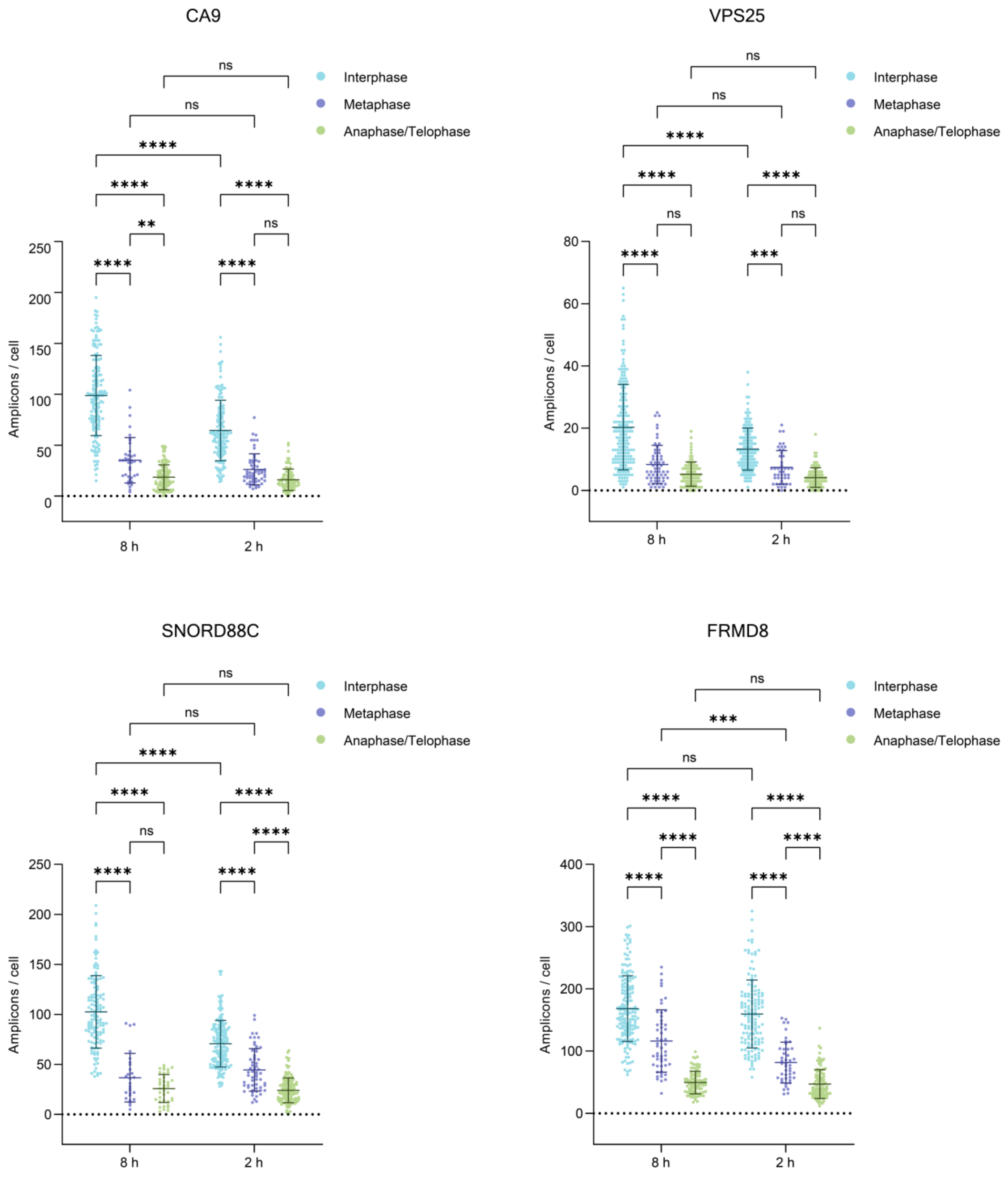

Figure S9. Two-way ANOVA of ac^{4}C RNA foci counts in HeLa cells.

HeLa cells were metabolically labeled with ethyl fluoroacetate for 2 h or 8 h. FMPLA was used to visualize and quantify ac^{4}C RNA foci corresponding to four RNA targets (CA9, VPS25, FRMD8, and SNORD88C) at different cell cycle stages, including interphase, metaphase, and anaphase/telophase. Two-way ANOVA was performed to evaluate the effects of labeling duration and cell cycle stage on ac^{4}C foci counts. Scatter plots show individual data points, with solid lines represent the median with quartiles. 2 h ethyl fluoroacetate labeling: CA9 Interphase: n = 150, mean ± SD = 64.4 ± 29.7; CA9 Metaphase: n = 58, mean ± SD = 26.2 ± 15.2; CA9 Anaphase/Telophase: n = 88, mean ± SD = 16.0 ± 10.4; SNORD88C Interphase: n = 160, mean ± SD = 70.7 ± 23.2; SNORD88C Metaphase: n = 59, mean ± SD = 44.5 ± 21.3; SNORD88C Anaphase/Telophase: n = 140, mean ± SD = 24.0 ± 12.4; VPS25 Interphase: n = 149, mean ± SD = 13.3 ± 6.74; VPS25 Metaphase: n = 45, mean ± SD = 7.38

± 5.39; VPS25 Anaphase/Telophase: n = 101, mean ± SD = 4.13 ± 3.15; FRMD8 Interphase: n = 141, mean ± SD = 159.6 ± 54.6; FRMD8 Metaphase: n = 45, mean ± SD = 81.6 ± 32.8; FRMD8 Anaphase/Telophase: n = 105, mean ± SD = 47.1 ± 23.1. 8 h ethyl fluoroacetate labeling: CA9 Interphase: n = 165, mean ± SD = 98.8 ± 39.3; CA9 Metaphase: n = 39, mean ± SD = 35.2 ± 22.3; CA9 Anaphase/Telophase: n = 121, mean ± SD = 18.4 ± 12.2; SNORD88C Interphase: n = 125, mean ± SD = 102.6 ± 36.2; SNORD88C Metaphase: n = 27, mean ± SD = 36.7 ± 24.3; SNORD88C Anaphase/Telophase: n = 39, mean ± SD = 25.9 ± 14.0; VPS25 Interphase: n = 185, mean ± SD = 20.3 ± 13.8; VPS25 Metaphase: n = 59, mean ± SD = 8.32 ± 6.15; VPS25 Anaphase/Telophase: n = 90, mean ± SD = 5.24 ± 3.89; FRMD8 Interphase: n = 163, mean ± SD = 168.2 ± 52.8; FRMD8 Metaphase: n = 52, mean ± SD = 116.3 ± 50.1; FRMD8 Anaphase/Telophase: n = 90, mean ± SD = 49.5 ± 18.1. ns, not significant, $P < 0.01$ (**), $P < 0.001$ (***) and $P < 0.0001$ (****).

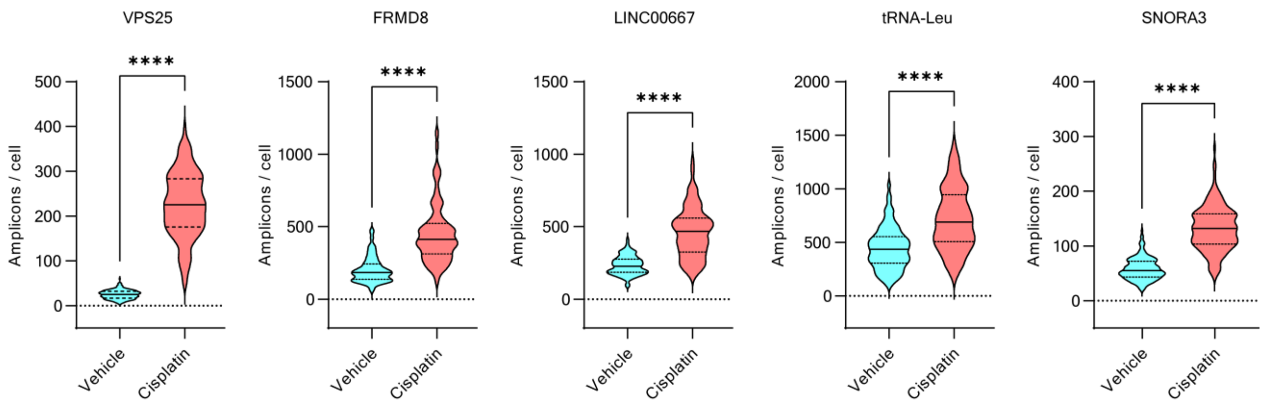

Figure S10. Quantification of RNA ac⁴C amplicons in the HeLa cells with and without cisplatin treatment.

Violin plots illustrating the distribution of fluorescent spots representing RNA ac^{4}C amplicons across HeLa cell groups treated with or without cisplatin for VPS25, FRMD8, LINC00667, tRNA-Leu, and SNORA3. Solid lines represent medians, while dashed lines indicate quartiles. VPS25 Vehicle: n = 199, mean ± SD = 25.0 ± 9.7; VPS25 Cisplatin: n = 128, mean ± SD = 225.4 ± 72.1; FRMD8 Vehicle: n = 191, mean ± SD = 202.8 ± 87.7; FRMD8 Cisplatin: n = 142, mean ± SD = 452.0 ± 203.4; LINC00667 Vehicle: n = 122, mean ± SD = 233.0 ± 64.2; LINC00667 Cisplatin: n = 115, mean ± SD = 461.7 ± 170.3; tRNA-Leu Vehicle: n = 195, mean ± SD = 443.0 ± 191.8; tRNA-Leu Cisplatin: n = 98, mean ± SD = 716.3 ± 287.9; SNORA3 Vehicle: n = 172, mean ± SD = 58.2 ± 19.9; SNORA3 Cisplatin: n = 164, mean ± SD = 131.7 ± 40.4. $P < 0.0001$ (****).

Table S1. The oligonucleotide sequences.

Oligonucleotide names	Oligonucleotide sequences (5' to 3')	Notes
Probe C1	Phosphate-CTATTAGCGTCCAGTGAATGCGAGTCTCGAGGTGCATTCATATCAGCAGCCGTCAAGAGTGTCTA	backbone
Probe C2	Phosphate-GTTCTGTCATATTTAAGCGTCTTAA	backbone
DBCO-Probe A	DBCO-AAAAAAAAAATATGACAGAACTAGACACTCTT	backbone
Probe C-Positive-1	GACGCTAATAGTTAAGACGCTT	positive control
Probe C-Positive-2	TATGACAGAACTAGACACTCTT	positive control
Probe B-Random	GACGCTAATAGTTAAGACGCTTAAAAAAAAAATTCCACCGCATGTCTACCATTC	random control
18s-1337-block	GCCATGCACCACCACCCACGGAATCGAGAAAGAGCTA	Blocking oligonucleotide
18s-1842-block	GAAACCTTGTTACGACTTTTACTTCCTCTAGATAGTC	Blocking oligonucleotide
MB	FAM-CGACGACTCGAGGTGCATTCATATTCGTCG-Dabcyl	signal probe
Probe B-18s-1337-0 nt	GACGCTAATAGTTAAGACGCTTAAAAAAAAAAGCCATGCACCACCACCCACGGA	RNA in situ hybridization (RISH) probe
Probe B-18s-1337-5 nt	GACGCTAATAGTTAAGACGCTTAAAAAAAAAAGCACCACCACCCACGGAATCGA	RNA in situ hybridization (RISH) probe
Probe B-18s-1337-10 nt	GACGCTAATAGTTAAGACGCTTAAAAAAAAAAACCACCCACGGAATCGAGAAAG	RNA in situ hybridization (RISH) probe
Probe B-18s-1337-15 nt	GACGCTAATAGTTAAGACGCTTAAAAAAAAAACCACGGAATCGAGAAAGAGCTA	RNA in situ hybridization (RISH) probe
Probe B-18s-1842-0 nt	GACGCTAATAGTTAAGACGCTTAAAAAAAAAAGAAACCTTGTTACGACTTTTAC	RNA in situ hybridization (RISH) probe
Probe B-18s-1842-5 nt	GACGCTAATAGTTAAGACGCTTAAAAAAAAAACTTGTTACGACTTTTACTTCCT	RNA in situ hybridization (RISH) probe
Probe B-18s-1842-10 nt	GACGCTAATAGTTAAGACGCTTAAAAAAAAAATACGACTTTTACTTCCTCTAGA	RNA in situ hybridization (RISH) probe
Probe B-18s-1842-15 nt	GACGCTAATAGTTAAGACGCTTAAAAAAAAAACTTTTACTTCCTCTAGATAGTC	RNA in situ hybridization (RISH) probe
Probe B-18s-1842-468 nt	GACGCTAATAGTTAAGACGCTTAAAAAAAAAAGGAATTAACCAGACAAATCGCT	RNA in situ hybridization (RISH) probe
Probe B-CA9-420	GACGCTAATAGTTAAGACGCTTAAAAAAAAAAGGCATTATTCTGGGGTTCTTGA	RNA in situ hybridization (RISH) probe
Probe B-CA9-381	GACGCTAATAGTTAAGACGCTTAAAAAAAAAAAACAGTAGGTAGATCCTCTAAC	CA9 distal probe
Probe B-GAPDH-22	GACGCTAATAGTTAAGACGCTTAAAAAAAAAACGAACAGGAGGAGCAGAGAG	probe targeting ac^4C-negative site
Probe B-GAPDH-45	GACGCTAATAGTTAAGACGCTTAAAAAAAAAACAAAAGAAGATGCGGCTGACTG	probe targeting ac^4C-negative site

Probe B-ASPH-61	GACGCTAATAGTTAAGACGCTTAAAAAAAAAAGAA GACTTCACCCGCCTGCCGG	RNA in situ hybridization (RISH) probe
Probe B-ASPH-1614	GACGCTAATAGTTAAGACGCTTAAAAAAAAAAGTA TCCCACGCCAAGGTCATTT	RNA in situ hybridization (RISH) probe
Probe B-ASPH-2692	GACGCTAATAGTTAAGACGCTTAAAAAAAAAATCC TAAATGAATTGCAGCGAGG	RNA in situ hybridization (RISH) probe
Probe B-SNORA3	GACGCTAATAGTTAAGACGCTTAAAAAAAAAATCA GCAGTTACTCTTCGAGGAC	RNA in situ hybridization (RISH) probe
Probe B-SNORD88C	GACGCTAATAGTTAAGACGCTTAAAAAAAAAAGGT GCACTGTGTCCTCAGGGGT	RNA in situ hybridization (RISH) probe
Probe B-LINC00667	GACGCTAATAGTTAAGACGCTTAAAAAAAAAAGGC GTGATTCTGGGAGGTCCAT	RNA in situ hybridization (RISH) probe
Probe B-FRMD8	GACGCTAATAGTTAAGACGCTTAAAAAAAAAAGCG CCCTCCCAGAGGTCAAGTT	RNA in situ hybridization (RISH) probe
Probe B-VPS25	GACGCTAATAGTTAAGACGCTTAAAAAAAAAAATC AGGAAGCTGGACTTGCTCT	RNA in situ hybridization (RISH) probe
Probe B-TOMM7	GACGCTAATAGTTAAGACGCTTAAAAAAAAAAGTC GGGAATCCGAAAGGGAAAG	RNA in situ hybridization (RISH) probe
Probe B-tRNA-Leu	GACGCTAATAGTTAAGACGCTTAAAAAAAAAAAGT CTGGCGCCTTAGACCACTC	RNA in situ hybridization (RISH) probe
siRNA-CA9+	GGAAGAAAUCGCUGAGGAATT	siRNA knockdown
siRNA-CA9-	UUCCUCAGCGAUUUCUUCCTT	siRNA knockdown
siRNA-NAT10+	GCAAUUGUACACAGUGACUTT	siRNA knockdown
siRNA-NAT10-	AGUCACUGUGUACAAUUGCTT	siRNA knockdown
CA9-FP	TATCTGCACTCCTGCCCTCT	RT-qPCR
CA9-RP	GGAATTCAGCTGGACTGGCT	RT-qPCR
NAT10-FP	AGTGGTCATCCTCCTACGGAC	RT-qPCR
NAT10-RP	TGTACCTGGAATGCACATCCAT	RT-qPCR
18S smiFISH-1	GCATGCCTACGTCGAACTCGTCGCAGTTTCACTGTA CCGG	smiFISH-First Strand Probe
18S smiFISH-2	GCATGCCTACGTCGAACTCGTAATCTTTGAGACAA GCATA	smiFISH-First Strand Probe
18S smiFISH-3	GCATGCCTACGTCGAACTCGTAACTGATTTAATGA GCCAT	smiFISH-First Strand Probe
18S smiFISH-4	GCATGCCTACGTCGAACTCGAATATACGCTATTGG AGCTG	smiFISH-First Strand Probe
18S smiFISH-5	GCATGCCTACGTCGAACTCGAGCTCTAGAATTACC ACAGT	smiFISH-First Strand Probe
18S smiFISH-6	GCATGCCTACGTCGAACTCGTCGTTATCGGAATTAA CCAG	smiFISH-First Strand Probe
18S smiFISH-7	GCATGCCTACGTCGAACTCGATCTAGAGTCACCAA AGCCG	smiFISH-First Strand Probe
18S smiFISH-8	GCATGCCTACGTCGAACTCGATCTGATCGTCTTCGA ACCT	smiFISH-First Strand Probe

18S smiFISH-9	GCATGCCTACGTCGAACTCGTGATAGGGCAGACGTTCGAA	smiFISH-First Strand Probe
18S smiFISH-10	GCATGCCTACGTCGAACTCGTCCATTATTCCTAGCTGCGG	smiFISH-First Strand Probe
18S smiFISH-11	GCATGCCTACGTCGAACTCGCAGCTAAGAGCATCGAGGGG	smiFISH-First Strand Probe
18S smiFISH-12	GCATGCCTACGTCGAACTCGTTAAAGTGGACTCATTCCAA	smiFISH-First Strand Probe
18S smiFISH-13	GCATGCCTACGTCGAACTCGAGATAGTCAAGTTCGACCGT	smiFISH-First Strand Probe
18S smiFISH-14	GCATGCCTACGTCGAACTCGTTCTCAGGCTCCCTCTCCGG	smiFISH-First Strand Probe
18S smiFISH-15	GCATGCCTACGTCGAACTCGTATGGTCGGAACTACGACGG	smiFISH-First Strand Probe
18S smiFISH-16	GCATGCCTACGTCGAACTCGATGCTTTCGCTCTGGTCCGT	smiFISH-First Strand Probe
18S smiFISH-17	GCATGCCTACGTCGAACTCGTGAGTCAAATTAAGCCGCAG	smiFISH-First Strand Probe
18S smiFISH-18	GCATGCCTACGTCGAACTCGATCTGTCAATCCTGTCCGTG	smiFISH-First Strand Probe
18S smiFISH-19	GCATGCCTACGTCGAACTCGCTAAGGGCATCACAGACCTG	smiFISH-First Strand Probe
18S smiFISH-20	GCATGCCTACGTCGAACTCGAAGTTTCAGCTTTGCAACCA	smiFISH-First Strand Probe
18S smiFISH-21	GCATGCCTACGTCGAACTCGAAACCATCCAATCGGTAGTA	smiFISH-First Strand Probe
18S smiFISH-22	GCATGCCTACGTCGAACTCGTATTGCTCAATCTCGGGTGG	smiFISH-First Strand Probe
18S smiFISH-23	GCATGCCTACGTCGAACTCGCTGGCAGGATCAACCAGGTA	smiFISH-First Strand Probe
18S smiFISH-24	GCATGCCTACGTCGAACTCGTTGATCTGATAAATGCACGC	smiFISH-First Strand Probe
18S smiFISH-25	GCATGCCTACGTCGAACTCGTCACCTCTAGCGGCGCAATA	smiFISH-First Strand Probe
secondary smiFISH-CY5	CY5-CGAGTTCGACGTAGGCATGC	smiFISH-Second Strand Probe

Table S2. The corresponding RNA types targeted by probe B, and their Accession Numbers.

Probe B Name	RNA	Accession Number
Probe B-18s-1337-0 nt	rRNA	NR_145820
Probe B-18s-1337-5 nt	rRNA	NR_145820
Probe B-18s-1337-10 nt	rRNA	NR_145820
Probe B-18s-1337-15 nt	rRNA	NR_145820
Probe B-18s-1842-0 nt	rRNA	NR_145820
Probe B-18s-1842-5 nt	rRNA	NR_145820
Probe B-18s-1842-10 nt	rRNA	NR_145820
Probe B-18s-1842-15 nt	rRNA	NR_145820
Probe B-18s-1842-468 nt	rRNA	NR_145820
Probe B-CA9-420	mRNA	NM_001216
Probe B-CA9-381	mRNA	NM_001216
Probe B-GAPDH-22	mRNA	NM_002046
Probe B-GAPDH-45	mRNA	NM_002046
Probe B-ASPH-61	mRNA	NM_004318
Probe B-ASPH-1614	mRNA	NM_004318
Probe B-ASPH-2692	mRNA	NM_004318
Probe B-SNORA3	snoRNA	NR_002580
Probe B-SNORD88C	snoRNA	NR_003069
Probe B-LINC00667	lncRNA	NR_015389
Probe B-FRMD8	miscRNA	XR_007062511
Probe B-VPS25	mRNA	NM_032353
Probe B-TOMM7	mRNA	NM_019059
Probe B-tRNA-Leu	tRNA	URS0000572B72_9606 (RNAcentral ID)
